# Combination Therapy Using Polyphenols: An Efficient Way to Improve Antitumoral Activity and Reduce Resistance

**DOI:** 10.3390/ijms231810244

**Published:** 2022-09-06

**Authors:** Alina Florentina Vladu, Denisa Ficai, Alexandra Gabriela Ene, Anton Ficai

**Affiliations:** 1 Department of Science and Engineering of Oxide Materials and Nanomaterials, Faculty of Chemical Engineering and Biotechnologies, University Politehnica of Bucharest, Gh Polizu St., 1-7, 010061 Bucharest, Romania; 2 National Centre for Micro and Nanomaterials, University Politehnica of Bucharest, Splaiul Independentei 313, 060042 Bucharest, Romania; 3 National Centre for Food Safety, University Politehnica of Bucharest, Splaiul Independentei 313, 060042 Bucharest, Romania; 4 The National Research and Development Institute for Textiles and Leather, Lucretiu Patrascanu, 030508 Bucharest, Romania; 5 Department of Inorganic Chemistry, Faculty of Chemical Engineering and Biotechnologies, Physical Chemistry and Electrochemistry, University Politehnica of Bucharest, Gh Polizu St., 1-7, 010061 Bucharest, Romania; 6 Academy of Romanian Scientists, Ilfov St. 3, 050042 Bucharest, Romania

**Keywords:** reactive oxygen species, oxidative stress, carcinogenesis, cancer therapy, synergistic effect, antioxidants

## Abstract

Polyphenols represent a structural class of mainly natural organic chemicals that contain multiple phenol structural units. The beneficial properties of polyphenols have been extensively studied for their antitumor, anti-inflammatory, and antibacterial effects, but nowadays, their medical applications are starting to be extended to many other applications due to their prebiotic role and their impact on the microbiota. This review focused on the use of polyphenols in cancer treatment. Their antineoplastic effects have been demonstrated in various studies when they were tested on numerous cancer lines and some in in vivo models. A431 and SCC13 human skin cancer cell lines treated with EGCG presented a reduced cell viability and enhanced cell death due to the inactivation of β-catenin signaling. Additionally, resveratrol showed a great potential against breast cancer mainly due to its ability to exert both anti-estrogenic and estrogenic effects (based on the concentration) and because it has a high affinity for estrogen receptors ERα and Erβ. Polyphenols can be combined with different classical cytostatic agents to enhance their therapeutic effects on cancer cells and to also protect healthy cells from the aggressiveness of antitumor drugs due to their anti-inflammatory properties. For instance, curcumin has been reported to reduce the gastrointestinal toxicity associated with chemotherapy. In the case of 5-FU-induced, it reduced the gastrointestinal toxicity by increasing the intestinal permeability and inhibiting mucosal damage. Co-administration of EGCG and doxorubicin induced the death of liver cancer cells. EGCG has the ability to inhibit autophagic activity and stop hepatoma Hep3B cell proliferation This symbiotic approach is well-known in medical practice including in multiple chemotherapy.

## 1. Introduction

Polyphenols represent a structural class of mainly natural organic chemicals that contain multiple phenol structural units. They can be classified into various subgroups depending on the number of phenol rings and based on structural elements that hold these rings together. Accordingly, the main classes of polyphenols are phenolic acids, flavonoids, stilbenes, and lignans. Cancer development is a complex process defined by three major stages: initiation, promotion, and progression. Initiation is a fast and irreversible step that can be generated by the uptake of, or exposure to, a carcinogenic agent, followed by its interaction with chromatin, leading to mutation or epigenetic modification [1]. 

Cancer patients first started to focus on natural products in their fight against the disease, mostly because of the numerous severe side effects and secondary toxicity induced by most conventional therapies [2]. At the same time, the pharmaceutical industry is testing new natural products that can be used in cancer treatment [2]. 

The advancements in cancer therapy were hampered by the appearance of drug resistance, high treatment costs, and increased reports of secondary toxicity, despite all the efforts made to raise awareness, early diagnosis, and new therapeutic interventions. In addition, known side effects commonly associated with the majority of chemotherapeutic drugs such as nausea, vomiting, headache, musculoskeletal pain, anorexia, gastritis, oral ulcers, diarrhea, constipation, alopecia, neuropathy, and so on, require additional therapies that further increase the treatment cost. The use of plants to fight cancer dates back several centuries, as reported in the ancient traditional folklore of Asia, Africa, and Europe. Various herb extracts and plant decoctions are considered to have the ability to prevent carcinogenesis, minimize tumor size, or remove cancer-related symptoms [2]. 

In this regard, natural products such as polyphenols may represent ideal alternatives, especially when administered with other drugs, where better efficacy and safety are necessary [3]. 

Plant polyphenols seem to be very promising antitumoral agents. Their antioxidant capacity has been strongly discussed in the literature, from additional in vivo studies performed in both rodents and humans, which showed low oxidative damage and high antioxidant capacity (ferric reduction plasma capacity, Trolox equivalent oxygen absorption capacity, etc.) both in the blood and tissues after administration [2]. 

Chemoprevention strategies in cancer seem to act through four major mechanisms:

1. By regulating inherited traits or modifications in gene expression determined by direct contact with carcinogens and that could initiate the carcinogenesis process;
2. By decelerating the effects of exacerbating factors (such as stress) that encourage the development of cancer cells;
3. By counteracting the survival of cancer cells through the regulation of cellular mechanisms;
4. By restricting the capacity of cancer cells to form metastases [2].

## 2. Reactive Oxygen Species in Cancer Development

Cancer has become one of the top-ranked public health issues worldwide, with millions of new cases each year. Even though the diagnosis tools, prognostic predictions, and conventional therapeutic strategies have reached a higher level in recent years, there are still some challenges that remain to be solved such as the high treatment costs and drug resistance. The number of deaths is still overwhelming, making cancer the second leading cause of death around the world, after cardiovascular diseases. Global cancer statistics (2018) have reported approximately 18.1 million cancer incidences and 9.6 million cancer-related deaths. Despite numerous attempts to improve the conventional therapeutic strategies in recent decades, only radiotherapy, chemotherapy, immunotherapy, and surgical techniques are clinically applied [4].

The risk of cancer continues to rise with the steady increase in life expectancy and harmful changes in eating habits, lifestyle, and environmental conditions [5]. Autophagy is a catabolic process that takes place inside the cell, where the dysfunctional cytoplasmic cargos are removed by the merging of cargo-containing autophagosomes with lysosomes to keep cyto-homeostasis. It has an important function in balancing the cytoprotective and cytostatic function in the case of malignant transformation. Antioxidant and immunomodulatory activity modulates autophagy and its signaling pathways with the purpose of stopping the incapacity of cells in the case of a metastatic process. Autophagy also modulates aberrant epigenetic regulation and inflammation, slowing down tumor metastasis [6]. 

Reactive oxygen species (ROS), typically produced by cellular metabolism, are involved in numerous cellular functions such as gene transcription, signaling transduction, and immune response. Nevertheless, the excessive production of ROS leads to the occurrence of various chronic and degenerative diseases. Therefore, high levels of ROS have been reported to take part in the initiation and progression of carcinogenesis. ROS interact with cellular compounds and act as cellular messengers, regulating various pathways including p53. This interaction can be directly involved in the etiology and progression of cancer. In physiological conditions, p53 reduces ROS generation and activates antioxidant genes, not allowing DNA damage and supporting cell survival. Additionally, p53 protein may inhibit NRF2 expression and activate pro-oxidant genes, leading to DNA damage and cancer cell apoptosis [3].

ROS are radicals that have a single unpaired electron in the outermost electron shell, which makes them highly reactive. ROS can be found either as free oxygen (such as superoxide, hydroxyl radical, nitric oxide, sulfoxyl radical, etc.) or as non-radical species (such as oxygen singlet, hydrogen peroxide, ozone, etc.). In the case of biological systems, superoxide, nitric oxide, hydrogen peroxide, and hydroxyl radicals are of the main interest. The major source of intracellular superoxide is the NADPH oxidase mediated oxidation of NADPH. Aerobic respiration also produces some amounts of superoxide. Inside the cell, superoxide is transformed into hydrogen peroxide under the mediation of superoxide dismutase. When the concentration of hydrogen peroxide is increased greatly, it reacts with metal cations and forms hydroxyl radicals that cause irreversible damage to nucleic acids and other macromolecules. This condition is called oxidative stress [7]. 

**Figure 1:**
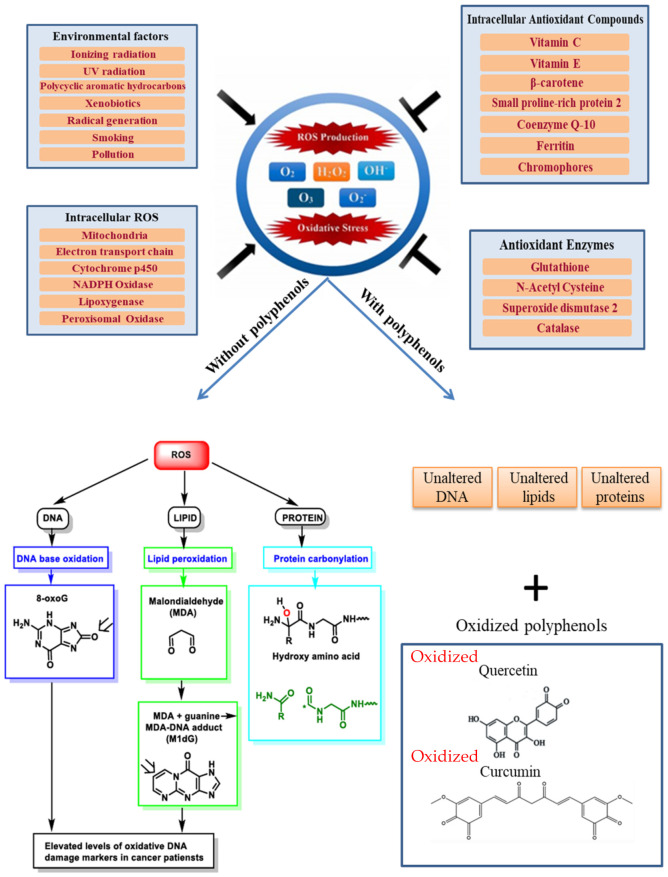
The ROS production, oxidative stress, and the balancing role of polyphenols, adapted according to [9,10].

Oxidative stress is a condition caused by the high generation of ROS together with the reduced antioxidant capacity of the cell (Figure 1). As previously mentioned, ROS are frequently produced in aerobic cells by the insufficient reduction of molecular O_2_ to H_2_O in the process of mitochondrial oxidative phosphorylation as well as during inflammation, mechanical and chemical stresses, infections, exposure to UV, and ionizing radiations. Different levels lead to different effects. Low ROS levels have beneficial effects by sustaining cell proliferation and survival pathways. On the other hand, high ROS levels can oxidatively damage biological macromolecules and lead to oxidative stress, which causes cell death. They can also lead to genotoxic damage such as single and double-strand breaks, modified bases, and DNA–protein cross-links. Cells are able to fight against oxidative stress by using various defense mechanisms such as free radical scavengers and antioxidant molecules. ROS are produced in larger amounts in highly proliferative cancer cells due to oncogenic mutations that support aberrant metabolism. ROS act as second messengers in intracellular signaling cascades, which maintain the oncogenic phenotype of cancer cells [8]. 

**Figure 2:**
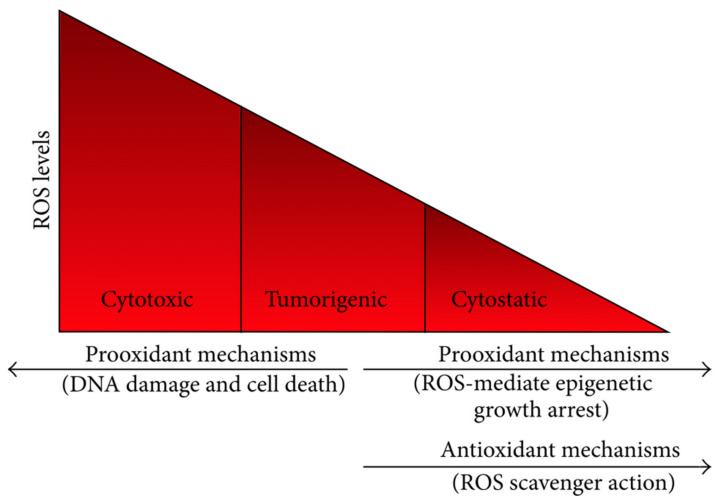
The dual prooxidant and antioxidant role of ROS levels in cancer cells [11].

It has been reported that prolonged inflammation may lead to a preneoplastic state. In cells suffering from chronic inflammation, the production of high amounts of ROS or reactive nitrogen species (RNS) attracts more activated immune cells that promote the amplification of dysregulated processes and the development of a preneoplastic situation. When the available endogenous antioxidant response is overcome by the ROS/RNS levels, irreversible oxidative deterioration of nucleic acids, proteins, and lipids may induce genetic and/or epigenetic alterations, resulting in the dysregulation of oncogenes and tumor suppressor genes. Therefore, the failure of overcoming the prolonged oxidative stress state and chronic inflammation may cause the initiation of carcinogenesis (Figure 2) [11]. 

For instance, a mix of green tea polyphenols and sulforaphane leads to the epigenetic reactivation of silenced tumor suppressor genes such as *p21^CIP/WAF1^* and KLOTHO through active chromatin changes in the breast cancer cell lines. This shows the potential of synergistical activity of the polyphenol-based therapeutic combinations [11].

The generation of ROS is involved in the modulation of chemotherapy or radiotherapy responses through its ability to downstream cell survival or death signaling cascades. This finding suggests that ROS modulators can be used for primary cancer prevention and to improve the therapeutic outcome [12]. 

ROS act as a ”double-edged sword” in oncogenesis. They can act as both tumor promoters and suppressors. The Ras-Raf-MEK-ERK signaling cascade can mediate the tumor-suppressive role of ROS. This pathway can stimulate the expression of p38 MAPK. p38 activation has been reported to be a prerequisite for ROS-mediated cancer cell apoptosis and inhibition or ROS clearance downregulating p38 dependent signaling. The activation of the p38 MAPK pathway that induces tumor suppression includes the DNA damage response, oncogene mediated senescence, and inflammation-induced cellular senescence [7].

**Figure 3:**
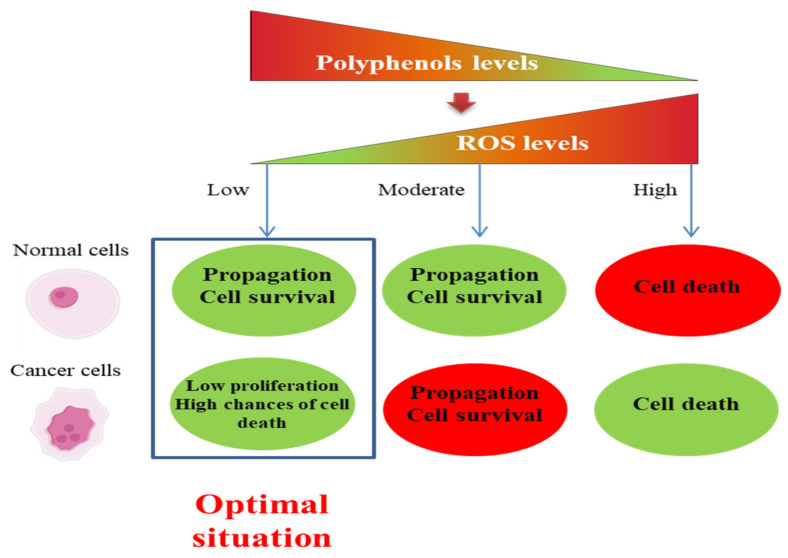
ROS as a “double edged sword” and the role of polyphenols [13].

ROS need to be kept at an optimum level to maintain cellular proliferation and survival. Increased ROS production and accumulation promote apoptosis. On the other hand, when ROS levels drop below a specific threshold, ROS-mediated signaling cascades can be disrupted and cell growth inhibited. Moreover, there are also high chances of cell death. By increasing cellular antioxidants, for instance, through polyphenol therapy and targeting sources of ROS, oxidative stress can be kept under control, leading to growth inhibition and high susceptibility to cell death in cancer cells, without affecting the normal cells very much. In this case, we can consider an optimal situation (tumoral cells are destroyed while normal cells are only marginally affected) (Figure 3) [13]. 

ROS can oxidize the nucleobases including guanine, leading to DNA damage. Errors can creep in during the repair processes of these modified bases, leading to mutagenesis. Radiation, for example, is one of the main sources of ROS and is associated with tumor-initiating events. ROS have important effects on protein function and therefore may adversely affect cellular processes. ROS produce effects that are closely related to the degree of protein oxidation. Mild oxidation is usually reversible (disulfides, sulfonic acid, sulfuric acid), allowing for quick modification in the protein activity and signaling pathways. On the other hand, excessive oxidation induces terminal oxidation (sulfonic acid) and irreversible loss of protein function. Irreversible cysteine changes can be harmful to protein function, while reversible changes can protect their function during stress. Protein changes are very important in the adaptation to oxidative stress through their activation of antioxidant or metabolic programs to promote ROS metabolism. The way that ROS act on tumor initiation and promotion is difficult to understand and is based on the amount, location, duration, and context [14]. 

The stage of tumorigenesis influences the effects of endogenous antioxidants. They can prevent tumor initiation by not allowing for the ROS-induced oxidation of DNA and subsequent DNA alteration. However, the direct role of antioxidants in preventing ROS-induced tumor initiation is not very clear [14]. 

Neoplastic mutations can happen during division when DNA is affected by ROS and cells containing such damaged, unrepaired, or misrepaired DNA. Mutations caused by ROS are typically base pair substitutions and less base insertions or deletions. ROS can affect all four types of nucleotide bases, however, GC base pairs are more affected than AT base pairs. Tumor initiation or progression can be explained by these types of mutations in oncogenes or tumor suppressor genes. For instance, G to T transversion is more common in the p53 gene in human cancers. It seems that there is also a correlation between redox imbalance, viral infection, and carcinogenesis. For example, chronic B and C hepatitis infection can disturb pro/antioxidant balance in the liver, leading to a higher level of pro-oxidants and the inhibition of antioxidant enzyme production. Therefore, oxidative stress can promote oncogenic transcription factors, resulting in hepatocellular carcinoma development [7]. 

The initial stages of tumorigenesis are characterized by a process called angiogenesis, which is when new blood vessels are formed from the pre-existing vasculature. This promotes tumor proliferation and survival, being known as the higher need of these cells. ROS-dependent angiogenesis is linked to cancer proliferation, increasing the metabolic rate, and resulting in the production of high ROS levels. The increased ROS levels promote oxidative stress in the tumor microenvironment, leading to the initiation of angiogenic modulator secretion [9]. 

It has been demonstrated that phytochemicals can act as chemo-preventive and synergistic agents that are able to enhance anticancer activity and simultaneously lower the chemotherapy-associated toxicity. Their pro-oxidative as well as antioxidative potency can modulate the apoptotic signaling in the direction of cancer prevention through the fact that they influence the homeostasis of ROS in a positive manner. Normally, the polyphenols can regulate apoptosis and autophagic cell death in cancer cells and, at the same time, inhibit these processes in normal organs around the tumor vicinity [4]. 

Considering all these positive aspects of plant-derived polyphenols in targeting ROS-modulated pathways, it seems that they could represent a promising weapon against cancer [3]. 

There is a complex integration of ROS type, level, and location according to the cellular responses to ROS. For instance, mitochondrial ROS are essential in promoting damage and death, whereas membrane generated ROS are usually considered to promote proliferation, migration, tumorigenicity, and metastasis. Still, these differences are not absolute, as mitochondrial ROS have been observed to also have a role in proliferation, migration, tumorigenicity, and metastasis, while NOX-generated ROS at the membrane could promote cell death through ferroptosis and necrosis [15]. 

Cancer cells are subjected to constant pro-oxidative stress. During the tumorigenesis process, sustained mitogenic signaling and oncogenic transformations increase the intracellular H_2_O_2_ in developing cancer cells. Furthermore, those highly proliferative tumors are able to overcome the vascular expenditure rate, making the regions inside the tumor suffer from hypoxia, therefore stimulating mitochondrial H_2_O_2_ generation. The levels of intracellular ROS are increased when tumor cells that proliferate outside of their matrix niches or that intravasate into circulation separate from the extracellular. Furthermore, blood and organs are particularly oxidative environments and increase the levels of ROS in circulating cancer cells [16]. 

Because of the increased generation of ROS in cancer, malignant cells are extremely dependent on antioxidant enzymes in order to manage the ROS stress. The constant oxidative stress that is caused by the presence of oncogenic signals and highly active metabolism presumably requires the entire utilization of cellular antioxidant capacity. It has also been presumed that an additional oxidative stress such as the exposure of malignant cells to ROS-generating anticancer agents may deplete the cellular antioxidant capacity and lead to a ROS stress level across the “threshold” that may induce apoptosis [17]. 

Phenolic compounds are a group of auxiliary metabolites in plant cells, counting flavonoids, tannins, lignins, and hydroxycinnamate esters. Phenolic acids also contain phenolic compounds such as antioxidants; these are the by-products of cinnamic acid benzoic acid, responsible for H_2_O_2_ scavenging. Anti-oxidative properties of polyphenols are based on [18]:

(1) Hydrogen or electron contributors donating high reactivity;
(2) Stabilization and delocalization properties to electron un-pairing;
(3) Chelating capacity for transmission metal ions;
(4) Membrane fluidity reduction.

One well-known natural polyphenol and autophagy inducer is resveratrol. It has been observed that it successfully suppresses NLRP3 inflammasome activation by lowering different activation signals such as ROS, mtDNA, and mitochondrial damage. Moreover, resveratrol controls mitochondrial ROS homeostasis by SIRT3 signaling and contributes to mitochondrial integrity through targeting SIRT1, which could be a plus for its ability to inhibit the inflammasome [6]. 

Another very interesting property of resveratrol is the ability to reverse the acquired resistance to classical chemotherapeutics in non-stem and stem cancer cells by sensitizing the cells in numerous ways. It has been shown that it can induce cancer cell apoptosis by being involved in pro- and anti-apoptotic factors; in the way that it interferes with drug and carcinogen-metabolizing enzymes; by regulating miRNAs; and by acting on drug resistance gene/protein expression and signaling pathways [19]. 

One particular study investigated the way polyphenols protect keratinocytes against UV-induced apoptosis. It has been observed that the p53 protein was overexpressed in extremely damaged keratinocytes by ROS, leading to apoptosis. Additionally, in the case of UVB-irradiated human skin, the treatment with lemon balm, black tea extracts, and resveratrol decreased oxidative stress, p53 expression, and the apoptosis in cell and animal models [3]. 

Quercetin has been reported to exert its protective property through the inhibition of survival and the induction of death signaling pathways in hepatocarcinoma. Quercetin in animal studies showed good protection against carcinogenesis due to its powerful antioxidant activity and subsequent prevention of mutations induced by ROS in the genes important for cell cycle control such as p53. There have been some concerns regarding the toxicity of quercetin, although human studies did not show adverse effects in the case of a single dose oral administration up to 4 g or 500 mg twice daily for one month [20]. 

As above-mentioned, the double-edged sword nature of polyphenols relies on the fact that even if a number of studies have reported the benefits of their antioxidant capacity for cancer therapy, there are still not enough solid trials on a large scale to confirm this aspect. Some contrasting results have shown that mice treated with vitamin E suffered from an increase in tumor development and metastasis, whilst another reported that increased vitamin C doses led to an increase in ROS levels able to determine the death of colon cancer cells holding KARS and BRAF mutations. Moreover, it seems that the administration of antioxidant N-acetylcysteine pushes the advancement of lung cancers and melanomas. Therefore, the results of various studies have led to the hypothesis that the inhibition of antioxidant enzymes assures the death of cancer cells, particularly when this method is used together with treatments that increase ROS. This method could represent an alternative to a traditional approach of targeting oncogenes and tumor suppressor genes, one that seems much more complicated because of the great number of genes involved. Furthermore, increased ROS-induced apoptosis has been observed in malignant cells after ATP consumption, resulting from the manipulation of glycolytic enzymes, radiation therapy, and chemotherapy [21]. 

## 3. Polyphenols as Anticancer Agents

Classical anticancer therapies usually induce a hostile cellular environment, leading to organelle impairment around the tumor and the development of drug resistance. To overcome such adverse effects, studies have started to focus more on the use of bioactive anticancer compounds due to their multi-target specificity, selectivity, and their cyto-friendly nature [6]. 

Anticancer drugs can stop the unusual proliferation of malignant cells, encourage the apoptosis of cancer cells, and decrease metastasis, all by targeting various molecules and signaling pathways. Thus, the finding of the anticancer potential of natural polyphenols has become a great interest for pharmacists. Resveratrol, for instance, is one of the most studied polyphenols due to its great anticancer activity. Many studies have reported that the anticancer effects of resveratrol are effective on all cancer development stages (initiation, promotion, and progression) and can intervene in several signaling pathways such as the activation of pro-apoptosis pathways (p53, Bax/Bcl-2), the resistance of cell cycle (Cyclin, P21), the inhibition of metastasis and angiogenesis related pathways (VEGF, TGF-, MMP), and the regulation of inflammatory responses (NFB, MAPK). Other polyphenols have also showed anticancer potential such as curcumin, (−)-epigallocatechin-3-gallate (EGCG), genistein, etc. Meanwhile, many studies have reported that an unusual tumor microenvironment (i.e., acidic pH, increased ROS levels, hypoxic conditions) has a huge impact on increasing the genetic instability and appearance of drug resistance. Hence, it is very important to manage the normalization of the malignant tissue microenvironment. The well-known antioxidant nature of polyphenols can properly modulate the tumor microenvironment. Moreover, as already mentioned, polyphenols can act as both antioxidants and pro-oxidants. If the concentration increases, hydroxyphenoxyl radicals are able to react with a second radical and generate toxic quinone, which can lead to covalent DNA damage. Additionally, polyphenols possess great photothermal characteristics, which are excellent in photothermal therapy (i.e., polydopamine) [22].

Table 1 shows the main properties of some polyphenols (EGCG, curcumin, caffeic acid, and resveratrol).

**Figure 4:**
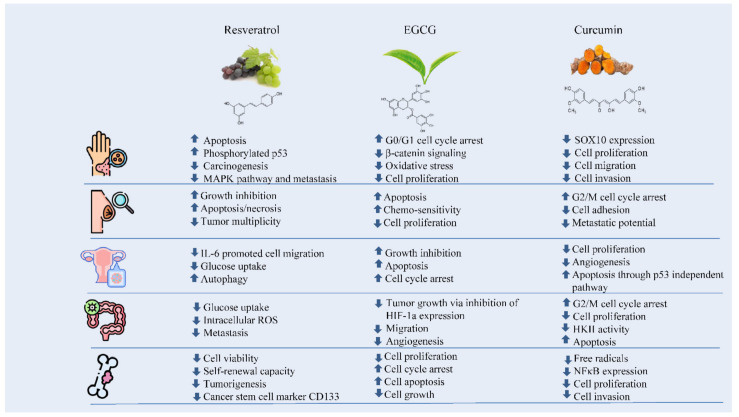
The effects of polyphenols against skin, breast, ovarian, colorectal, and bone cancers.

The numerous anticarcinogenic properties of polyphenols include their ability to suppress the formation of tumors, angiogenesis, metastasis, and inflammation as well as to trigger apoptosis. Additionally, they can control immune system responses and protect healthy cells from harmful free radicals. The majority of studies on the anticancer effects of polyphenols have been based on individual substances. For instance, resveratrol has been linked to a number of anti-cancer biological processes including the suppression of glucose uptake, metastasis, and the induction of apoptosis. It has been found that EGCG can control cancer cell growth, metastasis, angiogenesis, and other aspects of cancer evolution by altering several processes. Curcumin has also been shown to suppress cellular growth and angiogenesis, stop cell cycle progression in tumor cells, and trigger apoptosis in various cancer models (Figure 4) [24].

### 3.1. Skin Cancer

Skin cancers are the most frequent malignant neoplasm in humans, mostly in Caucasians. In the United States alone, more than two million people are annually diagnosed with non-melanoma and melanoma skin cancers. This means that the incidence of skin cancers is approximately equal to the combined incidence of cancers of all other organs. An important public health issue is represented by cutaneous cancers, which are a major health care expense. Even if sunscreens are used, they do not properly protect the skin against the damaging effects of solar ultraviolet radiation, which is an important cause of cutaneous malignancies. Therefore, it is highly necessary to design and develop functional therapeutic agents and more successful preventive approaches [5]. 

Skin possesses its own antioxidant protective mechanisms, which blocks some of the damaging effects of different carcinogens and environmental pollutants such as UV radiation, leading to the generation of oxygenated molecules called “free radicals”. However, when there is extensive exposure to these factors, the antioxidant capacity may be exceeded and become less efficient, causing premature aging, immunosuppression, and skin cancers. Prolonged carcinogen exposure can cause epidermal lipid peroxidation and unnecessary infiltration of the leukocytes into the skin. These can further cause the excessive production of hydrogen peroxide (H_2_O_2_), nitric oxide (NO), and other ROS, which leads to oxidative stress. Natural polyphenols protect cell constituents from oxidative damage by scavenging these free radicals [25]. 

The MAPK pathway consists of the extracellular signal-regulated kinase 1/2 (ERK 1/2), p38 proteins, and c-Jun-N-terminal-kinase (JNK). The activation of the MAP kinase pathway, mediated by the tyrosine kinase receptor, leads to the activation of transcription factor activator protein-1 (AP-1), which further activates the expression of MMPs. The p38 and JNK pathways are very important in increasing the expression of AP-1 and COX-2 mediated by UVA radiation and represent targets for skin cancer chemoprevention. The anticancer potential of polyphenols results from the inhibition of the MAPK pathway. For instance, black tea polyphenol and resveratrol reduced the expression of JNK, phosphorylated ERK 1/2, and p38 and enhanced apoptosis and phosphorylated p53 in the skin cancer cells, leading to the prevention of skin carcinogenesis. Resveratrol also inhibits cancer cell migration and metastasis through the inhibition of the MAPK pathway [26]. 

EGCG, a green tea polyphenol, has been reported to possess anti-carcinogenic properties on a number of skin tumor models, therefore, the focus has been on studying the molecular targets that are linked to its cytotoxicity against cancer cells. Cancer cell proliferation is accelerated by constant inflammation, which has been demonstrated to play an important role in β-catenin signaling activation. Due to recent observations of the fact that β-catenin is upregulated in skin cancer cells, it seems that the anti-skin carcinogenic properties of EGCG may be mainly mediated by its effect on β-catenin signaling [5]. 

Proliferation plays a huge role in cancer development and progression characterized by abnormal activity and the expression of cell cycle proteins. The cell cycle is the process of cell progression and division. Regulatory proteins involved in cell cycle are cyclines, CDK interacting proteins (CIPs) including p21, cyclin-dependent kinases (Cdks), kinase inhibitory proteins (KIPs) including p27, Cdk inhibitors (INKs) including p18, surviving and p53. Cancer is characterized by a poor functioning of these regulatory processes, which lead to unrestricted cell proliferation, and finally tumor growth and progression. EGCG has been reported to modulate the cell cycle through cell regulatory proteins, resulting in cell cycle arrest and decreased cellular proliferation. EGCG is able to arrest cells in the G0/G1 cell cycle phases, as long as its combination with other compounds has been demonstrated to induce G0/G1, G2/S, and G2/M cell cycle arrest in many cancer models [27]. 

Various polyphenols have been successfully used to inhibit TNF-α. For example, a polyphenol known as punicalagin, extracted from pomegranate, was used for the protection of human dermal fibroblasts from cell death caused by UV irradiation by downregulating NF-κB caspase-3 and upregulating the transition phase G0/G1 and the DNA repair process. EGCG and resveratrol are able to diminish UVB-induced ROS upregulation of TNF-α and IL-6, the levels of mRNA, and further inhibit NF-κB expression, leading to a general anti-inflammatory activity. Additionally, resveratrol inhibited the expression of transforming growth factor TGF-β2 caused by the skin cells’ exposure to UVB, which is linked to the blocking of TGF-β2/Smad-dependent and independent pathways [23]. 

Due to the benefits of polyphenols in in vitro and preclinical studies, clinical trials have also been conducted to reveal the protective activity of polyphenols in skin cancer. In a randomized clinical study, the antioxidative properties of mixtures made of tea polyphenols and milks were examined in 44 healthy subjects. It was observed that there was a reduced level of oxidative stress in the treatment group aside from the placebo group, which resulted in enhanced texture and integrity of thee dermis in young and aged subjects [23]. 

Moreover, it was observed that the A431 and SCC13 human skin cancer cell lines treated with EGCG presented a reduced cell viability and enhanced cell death, and as above-mentioned, these effects were due to the inactivation of β-catenin signaling. Additionally, EGCG seems to be able to exert a cytotoxic effect on malignant skin cells without a notable harm to healthy skin cells [5].

Studies have suggested that EGCG can also cause cell cycle arrest in A431 skin cancer cells by inhibiting Cip1/p21 with no other modifications in Kip1/p27, cyclin D1, and CDK2, but a decrease in CDK4 at low doses [27].

Aside from the topical application of polyphenols, dietary ingestion of grape seed extract has been reported to bring many benefits in avoiding DMBA-induced TPA promoted two-stage skin carcinogenesis, slowing down the malignant transformation of papillomas into carcinomas, diminishing DMBA-induced inflammatory hyperplasia and reducing the proportion of mice with codon 61 and Ha-ras oncogene mutations. Studies have suggested that topical as well as the dietary feeding of grape seed extracts (resveratrol, quercetin, catechin) had beneficial results in reducing DMBA-induced epidermal hyperplasia, inflammation, and proliferation. The concomitant oral and topical administration have been demonstrated to be more effective than separate use and lead to lower inflammation, oxidative stress, and mutations of Ha-ras in codon 61 [28]. 

Sticking to grape seed polyphenols, it has been demonstrated that SKH-1 hairless mice fed with grape seed proanthocyanidins presented a reduced tumor incidence, size, and proliferation in the complete stages (both initiation and promotion) of UVB-induced photocarcinogenesis. Moreover, the red grape seed extract showed great efficacy in preventing UVB-induced oxidative stress when applied directly on the skin by enhancing the levels of GSH and glutathione peroxidase through the inhibition of lipid peroxidation and nitric oxide generation. Additionally, grape seed polyphenols decreased the UVB-induced infiltration of proinflammatory leukocytes and reduced myeloperoxidase, prostaglandin, cyclooxygenase-2, cyclin D1, and proliferating cell nuclear antigen activities in skin tumors [28]. 

Toll-like receptor 4 (TRL4) seems to have a high importance in melanoma and tea polyphenols have a great anticancer activity. Thus, Chen et al. investigated the way that the tea polyphenols act on melanoma cells. In the study, tea polyphenols and lipopolysaccharides (LPS) were used to treat the B16F10 and A375 melanoma cell lines. Tea polyphenols reduced the proliferation, migration, and invasion capacity of cancer cells in a time and dosage dependent manner. It was observed that TRL4 was highly expressed in the skin cancer cells compared with the healthy skin cells. Tea polyphenols were able to inhibit TRL4 expression in both the stimulated and normal melanomas via the TRL4 antagonist LPS. TRL4 suppression can reduce cell function, therefore tea polyphenols have the ability to reduce melanoma growth in vivo [29].

### 3.2. Breast Cancer

Breast cancer represents one of the top causes of death in women worldwide. In a statistic from 2018 by the American Cancer Society, it was reported that around 30% of all new cancer cases in women were breast cancer and caused 40,920 deaths in the USA alone. It appears that one in eight women will suffer from breast cancer and the WHO reports show that the incidence will continue to raise. Around 80% of entire breast cancer cases diagnosed in postmenstrual females are estrogen receptor alpha positive, which means that they are very influenced by the presence of estrogen. Additionally, estrogen activity plays a highly important role in breast cancer prevention and therapy [30]. 

Resveratrol is a polyphenol usually found in grapes and red wine and has various health benefits. It has many great properties such as anticancer, neuro protective, anti-aging, antimicrobial, and anti-inflammatory. It has been reported that resveratrol is extremely beneficial against breast cancer mainly due to its ability to exert both anti-estrogenic and estrogenic effects (based on the concentration) and because it has a high affinity for estrogen receptors ERα and Erβ. At a concentration of 50 μM, it exerts anti-estrogenic effects in order to inhibit cell migration while, at a concentration of 5 μM, it exerts estrogenic effects by enhancing the invasion, migration, and development of lamellipodia on the ERα (−), ERβ (+) MDA-MB-231 breast cancer cell line. Lamellipodia is represented by some actin structures that can be observed at the leading edge of migrating cells, which are regulated by Rac. A total of 5 μM of resveratrol enhances Rac activity while 50 μM of resveratrol inhibits its activity in breast cancer cells. Concentration is also important in the case of Akt and MAPK. Increased concentrations reduce their activity while low concentrations have been reported to support proliferation in cancer cells and enhance Akt and MAPK activities, together with some other tumorigenic signaling proteins. Hence, the development and metastasis of breast cancer can be controlled based on the resveratrol dose [31].

Resveratrol is also a cycloocygenase-2 (COX-2) inhibitor, meaning that it has excellent anti-inflammatory properties. Considering all of the great properties exerted in human health, the addition of resveratrol in various mixtures has become very popular. COX inhibition and antioxidative activity are among the properties that participate in the commonly acknowledged anticancer and chemopreventive effects of resveratrol against a variety of malignancies including breast cancer. These mechanisms are able to keep the DNA away from oxidative damage and decrease prostaglandin-induced cancer cell proliferation. Moreover, it seems that resveratrol can also block other enzymes that participate in carcinogenesis and tumor progression such as ribonucleotide reductase, ribonuclease and human DNA ligase, RNA and DNA polymerases. Additionally, various in vitro studies have shown that resveratrol modulates gene expression and promotes the apoptosis of cancer cells via the downregulation of TP53, NF-κB, and Bcl-2, which are well-known transcription factors involved in tumor growth promoting gene activation [30].

One potential explanation regarding the double effect of resveratrol on Erα+ breast cancer cells is linked to the structural similarity of resveratrol with E2, which could mediate an interaction between resveratrol and both ERs, leading to estrogen-like effects and enhancing cancer cell proliferation. Although considering that the binding affinities of resveratrol to ERα and ERβ are as low as 0.0087 and 0.0102%, respectively, in comparison to original E2, there seem to be other mechanisms that are involved in the tumor growth inducing effects of resveratrol, especially at concentrations of less than 10 μM. A clinical study performed on 40 healthy female subjects showed that a daily high dose oral administration of 1.0, 2.5, or 5.0 g of resveratrol for 29 days led to high values of plasma levels far above the concentration needed to block the estrogen metabolism (0.62, 1.45, or 4.24 μM) [30].

Statistics show that HER-2 positive breast cancer is among the most aggressive subtypes and is responsible for approximately 30% of diagnosed cases, being associated with tumor invasiveness, low disease-free survival, and bad overall prognosis. Additionally, many patients have started to develop resistance to classical therapies. Thus, various current clinical trials are investigating new possible therapies. More and more studies have shown that polyphenols have great potential in breast cancer prevention and treatment, directly or indirectly via epigenetic regulation (i.e., micro RNAs) [32]. 

EGCG has been reported to be the only polyphenol that is found in plasma at high levels (77–90%) in free form. Its constant administration seems to help with breast cancer prevention by promoting apoptosis and reducing cell proliferation. For example, EGCG particularly induces cell growth inhibition by reducing HER2 and STAT3 phosphorylation in HER2 overexpressing BT474 breast cancer cells. When Her-2 positive breast cancer cells (AU565 and MCF-7) were exposed to EGCG, the inactivation of the PI3K/Akt and MAPK cascade signaling, the suppression of heregulin-b1-induced fatty acid synthase expression, and high caspase-9 activity were observed. Moreover, at even higher concentrations, EGCG enhanced the treatment sensitivity of trastuzumab-resistant HER-2 positive breast cancer cells via an increased apoptotic rate and reduced Atp production and cell growth [32]. 

Another polyphenol that has shown high efficacy against HER2 breast cancer cells is curcumin. It has been observed that it induced apoptosis by raising the BAX/BCL-2 ratio in the case of cells treated with 6–50 μM for 24 or 48 h. Furthermore, curcumin is much less toxic compared to classical chemotherapies. For instance, in the case of BALB-neuT transgenic mice that were administered 2 mg of curcumin in 50 μL of corn oil, three times a week for 14 or 24 weeks, a reduction in tumor proliferation and better tumor-free survival were observed, everything without potential side effects [33]. Studies linked to the second-generation curcumin analog RL66 reported excellent anti-tumorigenic potential in HER2 overexpressing SKBR3 breast cancer cells. When 1–3 μM of RL66 was used, it was observed that after 12–36 h, the intrinsic apoptosis was triggered, along with cell cycle arrest and reduced Her2 phosphorylation [32].

In addition, the Mediterranean diet, which includes various bioactive components, has been addressed in cancer treatment. Vinod et al. [34] demonstrated, on HER2 overexpressing SKBR3 breast cancer cells, that resveratrol had the ability to cancel docetaxel-associated HER2 phosphorylation, along with further activation of its related-downstream (MAPK and Akt) signaling cascades, at a concentration of 10–25 μM resveratrol and 0.1–10 nM docetaxel. The synergistic use of resveratrol and docetaxel showed enhanced cytotoxicity, which led to an increased apoptosis rate due to caspase-8,-9,-7,-3 activation and the break in their downstream target protein, PARP. Apoptotic cell death was possible due to the gathering of cells in the sub-G0 phase and DNA disruption. Additionally, the activation of Akt, ERK, Bad, JNK, BCl-2, and P38 under docetaxel activity was stopped by resveratrol pre-treatment, along with the nuclear translocation and DNA binding of Ap-1. It has also been reported that Akt offers resistance to docetaxel. The ability of resveratrol to act synergistically and as a chemosensitizing agent is linked to AKT2 downregulation by resveratrol and to the reduction in Akt-targeted anti-apoptotic protein survival and gene XIAP (which offers taxane-resistance because it promotes a premature mitotic exit [32]. 

Another study by Luo et al. described that EGCG, when used with paclitaxel, acts in a synergistic way by sensitizing cancer cells both in vitro and in vivo. An excellent reduction in the proliferation and an enhancement in taxol-induced apoptosis were reported in various breast cancer cell lines in vitro due to the ability of EGCG to increase the activation of c-Jun N-terminal kinases (JNKs) mediated by paclitaxel. Its sensitizing ability was also observed in vivo through the growth inhibition of 4T1 breast cancer cells in mice [35,36]. 

Another polyphenol that is able to potentiate epirubicin-induced apoptosis in MDA-MB-231 breast cancer cell is ferulic acid. This activity may be regulated through the Bax/Bcl-2/Caspase-3 pathway and PDI/IRE1α/PERK module of the endoplasmic reticulum stress signaling pathways. The results showed that the combined activity of ferulic acid and epirubicin enhanced the level of expression of endoplasmic reticulum stress proteins (PDI, PEPK, and IRE1α). These findings suggest that ferulic acid can act as an adjuvant in breast cancer treatment [37].

### 3.3. Ovarian Cancer

Ovarian cancer is considered as the deadliest cancer in females. The main problem associated with ovarian cancer treatment is the development of chemo-resistance. Similarly, with other cancer types, studies have focused on finding combined therapeutic strategies such as the administration of natural products. Studies have been performed on the effects of grape seed extract on the OVCAR-3, chemo-resistant cell line, and showed that it can inhibit cell growth and proliferation, while promoting the apoptotic process. The anti-proliferative activity of grape seed extract may be due to an increase in PTEN and DACT1 gene expression as well as the inhibition of the PI3K/AKT/MTOR and Wnt/β-catenin signaling pathway. Furthermore, the grape seed extract may destroy ovarian cancer cells by encouraging both extrinsic and intrinsic apoptotic pathways [38].

Additionally, resveratrol specifically acts as a multi-targeting drug by regulating signal transduction pathways that influence cell cycle progression proliferation, inflammation, metastasis, apoptosis, and angiogenesis. In the particular case of ovarian cancer, resveratrol led to a cessation of interleukin IL-6-promoted cell migration via ARH-1 activation, which is a tumor suppressor that modulates autophagy promotion [6,39].

Additionally, a number of in vitro studies have pointed out the inhibitory role of resveratrol on cellular glucose metabolism. When tested on ovarian cancer cells, resveratrol reduced the use of glucose and induced autophagy, emphasizing the conditions of nutrient deprivation [40].

**Figure 5:**
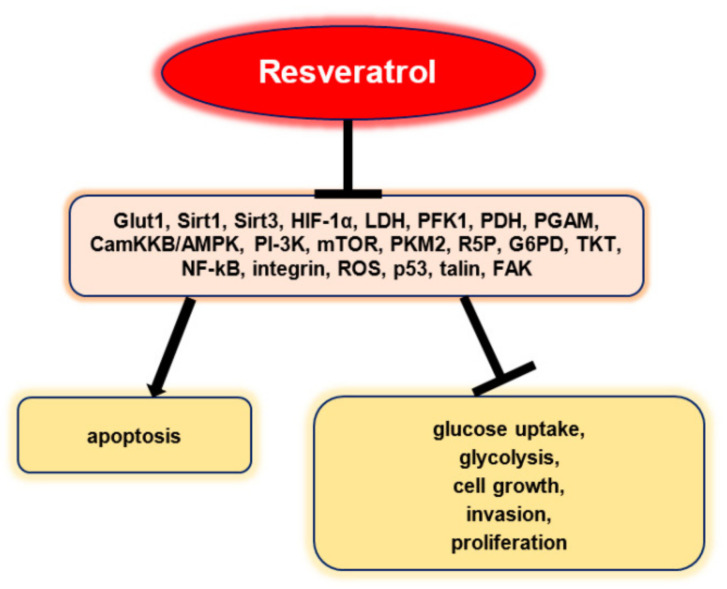
Resveratrol’s anti-neoplastic effects via the regulation of tumor glucose metabolism [39].

Resveratrol inhibited glucose uptake, glycolysis, cell growth, invasion, and proliferation in a selective manner and promoted apoptosis without being influenced by p53 status in vitro (Figure 5). Resveratrol did not affect mRNA, GLUT1, and protein expressions, but stopped intracellular GLUT1 to reach the plasma membrane. This effect seems to be associated with the inhibitory effect of resveratrol on Akt activity. Therefore, these results highlight the fact that resveratrol can promote the apoptosis of ovarian cancer cells by affecting glucose uptake, a process that involves Akt-regulated plasma membrane GLUT1 trafficking [41].

In vivo, resveratrol led to reduced glucose uptake in a mouse model, showing antineoplastic properties and the inhibition of tumor regrowth after cytostatic therapy (cisplatin) [42].

Regarding EGCG utilization, it was observed that this polyphenol enhanced the anticancer capacity of chemotherapeutic compounds in ovarian cancer. Chen et al. described that EGCG potentiated cisplatin susceptibility and reduced ovarian cancer cell growth via hydrogen peroxide (H_2_O_2_) delivery. This polyphenol potentiated the beneficial results of cisplatin up to six-fold in SKOV 3, CAOV3, and C200, a cisplatin-resistant ovarian cancer cell line. Its efficacy could be associated with the fact that it can increase intracellular H_2_O_2_ levels, meaning that higher oxidative stress could enhance the chemotherapy efficacy in ovarian cancer [35,43]. 

Another study by Yallapu et al. analyzed the way curcumin-based nanoparticles influence A2780CP cisplatin resistant ovarian cancer cells. Therefore, in order to enhance the curcumin pharmacokinetics in vivo, curcumin nanoparticles were conjugated with a monoclonal antibody with an affinity for tumor cells. These nanoparticles had excellent results in inhibiting the proliferation of A2780CP ovarian cancer cells, suggesting that this formulation could improve curcumin release to the tumor site and sensitize radio- and/or chemo-resistant cancer cells with high specificity [35,44]. 

The aflavin-3,3′-digallate (TF3) is another polyphenolic agent extracted from black tea that has shown great potential against ovarian cancer cells. As above-mentioned, this type of cancer has a low survival rate because cells usually develop cisplatin resistance. The research by Pan et al. investigated the synergy between TF3 and cisplatin in the A2780/CP70 and OVCAR3 cells. A combined pro-apoptotic effect and the arrest of cells in the G1/S phase have been observed. In addition, there was a regulation of the protein expression of cytochrome c, cleaved caspase 3/7, Bcl-2, and Bax. The synergistic use led to G1/S cell cycle arrest by the modulation of cyclin A2, D1, E1, and CDK2/4 protein expression. Additionally, the simultaneous use of these two compounds could downregulate Akt phosphorylation in both cell lines [45].

EGCG, the main component of green tea, is able to strongly bind to small molecular drugs, proteins, and DNA. Thus, it can be used for biomacromolecules and drug delivery. Chuan et al. developed a drug delivery system based on polyethylene glycol folic acid functionalized EGCG and doxorubicin for the targeted therapy of ovarian cancer. Studies have revealed that the system improved doxorubicin uptake by the SKOV3 cancer cells when compared with the system without further folic acid functionalization. Additionally, the in vitro tests showed higher toxicity and tumor growth inhibition for the folic acid functionalized system on SKOV3 cells [46].

Green tea and paclitaxel are another combination that has shown improved anti-neoplastic activity in ovarian cancer when compared with the separate effects of the two agents. The synergistic effect acts by the inhibition of Akt phosphorylation. Many studies have demonstrated that increased Akt signaling pathway activation leads to a lower apoptosis rate in multiple cancer types via phosphorylation and the inactivation of pro-apoptotic mediators such as the Bad protein [47]. The inhibition of the Akt pathway was associated with the activation of the mitochondrial apoptotic pathway characterized by an important reduction in the anti-apoptotic BCL-2 protein and a notable increase in the Bad levels, Cyt-c, cleaved-caspases-3 and -9, and Bax [48].

### 3.4. Colorectal Cancer

In 2008, the American Cancer Society stated that colorectal cancer was the third most common cancer type in Western countries and has caused around 10% of all cancer related deaths in the U.S. A concerning increase in areas that were previously at low risk such as Africa, Asia, and Latin America has also been observed. Statistics worldwide have reported differences in the incidence, showing that environmental factors play a huge role in disease development. Some of the factors that may influence colorectal cancer development are obesity, diets high in calories, and sedentariness. There are clues that indicate that the mechanism of these risk factors is controlled by hyperinsulinemia and that insulin could enhance the growth of colon tumors [49].

The antitumoral activity of resveratrol was investigated in the HCT116 and Caco 2 human colorectal cancer cell lines. The results showed that resveratrol had the ability to inhibit the proliferation of both HCT116 and Caco2 colon cancer cells and to reduce pyruvate kinase and lactate dehydrogenase glycolytic enzymes in Caco2 cells. At the same time, an enhancement in the citrate synthase activity and a reduction in glucose expenditure were reported in both cell lines. Additionally, resveratrol managed to downregulate leptin and c-Myc expression and reduce the quantity of VEGF (vascular endothelial growth factor). An activation of caspases 3 and 8 apoptotic markers and an increase in the Bax/Bcl-2 ratio was observed. The study suggested that the calorie-restriction pathway may be the cause of this activity [49].

Resveratrol loaded polyethylene glycol–polylactic acid polymeric nanoparticles were tested both in vitro and in vivo on colon cancer and they led to delayed tumor growth together with an increased survival rate. The study suggested that the antitumoral and metabolic effect of resveratrol were preserved by polymeric nanoparticle loading both in vitro and in vivo [50].

Additionally, resveratrol changes the lipidomic profile, acts on raising the oxidative capacities via the CamKKB/AMPK pathway, and reduces glycolysis, along with a reduced pentose phosphate activity and higher ATP production in the HTC116 and Caco2 colon cancer cells [51].

Resveratrol also inhibits the glycolysis and glucose uptake in HT-29 cells. This metabolic response relies on the ability of resveratrol to inhibit intracellular ROS, which further downregulates HIF-1α accumulation, glycolytic flux, and Glut-1 expression [40].

Like any other type of cell, cancer cells demand a continuous flux of nutrients to properly grow and divide. Therefore, when the blood supply requirement is not fulfilled, the cancer will eventually stop growing. Angiogenesis represents the physiological process that leads to the formation of new blood vessels from pre-existing ones. Cancers stimulate angiogenesis through the secretion of several growth factors such as VEGF, which plays the main role in angiogenesis. When cancer angiogenesis is inhibited, the malignant tissue will die. Moreover, there are some factors that play important parts in metastasis: cancer cell mobility, migration, and invasion. Hence, inhibiting at least one of these three factors will stop metastasis. EGCG showed excellent potential in reducing angiogenesis, cell mobility, migration, invasion, and metastasis markers in various human cancers. In colorectal cancer, EGCG stopped tumor growth in SW837 cells in vitro and also in vivo. This happened via activation of the VEGF/VEGFR axis through the inhibition of HIF-1a expression and some other important growth factors. These polyphenols also reduced the migration and proliferation in SW620 cells in vitro via suppression of the PAR2-AP and VIIa factor together with the ERK 1/2 and NF-jB pathways [27]. 

Honey polyphenols are also great antioxidants and have powerful anticancer properties. Cinaciosi et al. studied the activity of Manuka honey on cancer stem cells such as from colorectal cancer (HCT116 cell line) enriched by the in vitro sphere-forming assay. It has been observed that Manuka honey decreased the volume of the entire culture spheroids, modifying their morphological parameters and promoted the apoptosis and intracellular ROS increase in these cells. Moreover, it reduced the mRNA expression ABCG2—an ABC transporter and influenced the self-renewal ability via downregulation of the mRNA expression of one of the receptor membranes of the Wnt/β-catenin pathway [52].

Resveratrol was studied in vitro on AK4-knockdown colon cancer cells (SW480 and SW620) and showed that it could diminish the invasion and metastasis of colon cancer cells by reversing the expression of EMT (epithelial–mesenchymal transition) markers via the AKT/GSK-3β/Snail pathway. Actually, AKT1 can act as an important regulator of EMT colon cancer cells and be a possible therapeutic target for colon cancer [53,54].

**Figure 6:**
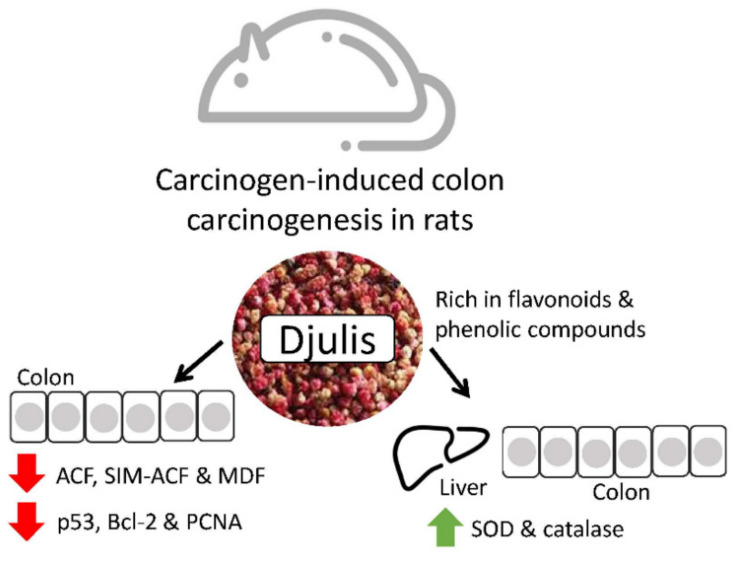
The effects of djulis in colon and liver cancer [55].

Djulis is a cereal that contains many polyphenols and fibers that have been reported to prevent colon malignancies. Lee et al. studied its effects on rats and discovered that the polyphenol content could reduce oxidative stress and modulate proteins involved in anti-apoptosis, pro-apoptosis, and proliferation to avoid colorectal cancer progression. Hence, in the future, djulis may be a great colorectal cancer chemopreventive product. Djulis can inhibit the generation of colonic preneoplastic lesions (ACF and MDF) in DMH-induced colon carcinogenesis in rats. In the colon of rats, djulis simultaneously raised the expression of proapoptosis-related proteins (Bax and caspase-9) and the activity of antioxidant enzymes (CAT and SOD). Additionally, p53, a protein related to proliferation (PCNA) and a protein connected to the prevention of apoptosis were all suppressed by Djulis (Bcl-2) (Figure 6) [54,55].

### 3.5. Osteosarcoma

Osteosarcoma is the most common primary malignant bone tumor in children and adolescents. The current treatment of osteosarcoma consists of surgical resection, radiotherapy, and chemotherapy. There are several existing drugs (salinomycin, cisplatin, doxorubicin, methotrexate, 5-fluorouracil, oxaliplatin, etc.) that are used for osteosarcoma treatment, but they have strong adverse effects and are effective when used in the initial stages. Therefore, more natural compounds with less side effects and lower toxicity on normal cells could be a great alternative, especially if synergies can be developed. One of these compounds is curcumin, which has been investigated for its pleiotropic effects, antioxidant, anti-inflammatory, antibacterial, and pro-wound healing activity, and for its ability to form curcumin–metal complexes [56].

Curcumin, a well-known polyphenol that has great antitumoral properties, was loaded in biodegradable copolymer coatings (polyvinyl alcohol-polyethylene glycol) through the MAPLE technique (matrix assisted pulsed laser evaporation) and tested on MG-63 cells, suggesting an improved reduction in osteosarcoma cell viability and proliferation [56].

Oxidative stress has various negative effects including osteoblast cell differentiation via the reduction of alkaline phosphatase RUNX2 differentiation markers and colony forming unit formation. Thus, antioxidant compounds such as polyphenols could act in a positive manner by protecting bone metabolism through the stimulation of osteoblast differentiation and the limitation of bone resorption. These protective properties have also been accomplished by other polyphenols such as EGCG and genistein. The loading of polyphenols and the optimization of delivery methods to the host tissue are important elements that influence the compound efficacy [57]. 

The normal process of bone reconstruction is characterized by continuous and alternate formation and resorption processes that are influenced by several molecular signaling pathways. However, the uneven effects of factors in these pathways caused reduced osteoblast activity and enhanced osteoclast activity. This means a disruption in bone formation and resorption, which leads to deficient bone regeneration. Many studies have demonstrated that polyphenols are important in the modulation of bone regeneration, primarily due to their antioxidant properties, which reduces the inflammatory response and stimulates the normal process of bone regeneration. Moon et al. reported that curcumin at a concentration of 5 μM is able to successfully clean free radicals and downregulate NFκB expression (the most important transduction factor involved in inflammation). Polyphenols can also influence the activation and modulation of antioxidant enzymes such as superoxide dismutase, catalase, and glutathione peroxidase, the main protector against ROS. The direct antioxidant activity, together with the stimulation of antioxidant enzymes, could successfully clean the existing ROS and prevent mitochondria from generating more free radicals. This helps with inhibiting osteoblast apoptosis via the suppression of the p53 apoptotic signal [22].

Nani et al. investigated the pro-apoptotic properties of *Pennisetum glaucum*, a pearl millet phenolic compound (PGPC), on U-2OS osteosarcoma cells. PGPC led to U-2OS cell death, proportional to the dose. PGPC downregulates AKT downstream and upstream effectors that are related to SAPK/JNK and p38 upregulation and high [Ca^2+^], leading to cell cycle arrest and caspase-dependent apoptosis in U-2OS osteosarcoma cells [58].

It has been demonstrated that resveratrol reduces the cell viability, self-renewal capacity, and tumorigenesis of osteosarcoma cells, while not causing any harm to normal osteoblast cells. Resveratrol also reduced the cytokine synthesis and blocked the JAK2/STAT3 signaling pathway, which influenced the reduction in the cancer stem cell marker, CD133. The obtained data showed that resveratrol stopped osteosarcoma cell proliferation and tumorigenesis capacity, which was linked to the cytokine inhibition related JAK2/STAT3 signaling blockage [59].

MicroRNAs represent a category of short noncoding RNAs and are strongly involved in gene regulation, pathogenesis, and human cancer progression. Zhu et al. studied EGCG activity against osteosarcoma. The analysis of cellular function reported that EGCG could reduce cell proliferation, promote cell cycle arrest, and induce osteosarcoma cell apoptosis in vitro, while also stop transplanted tumor growth in vivo. A series of analyses such as RT-qPCR and miRNA microarrays were performed and revealed that miR-1 was strongly upregulated in U-2OS and MG-63 cells treated with EGCG in a direct correlation with time and dose. The miR-1 downregulation by the inhibitor resembles the attenuated EGCG-induced inhibition on osteosarcoma cell growth. It was established that miR-1 was often reduced in clinical osteosarcoma tissues. Additionally, EGCG and miR-1 mimicked the inhibited c-MET expression, and mixt treatment with EGCG and c-MET inhibitor (crizotinib) improved the inhibitory effect on U-2OS and MG-63 growth. These results show that EGCG could have anticancer activity on osteosarcoma cells via the regulation of miR-1/c-MET interaction [60].

In the case of osteosarcoma, various studies have demonstrated the benefits of curcumin, which may stimulate U-2OS, MG-63, and HOS cells apoptosis based on several signaling pathways. Moreover, curcumin has also been reported to inhibit proliferation, invasion, and metastasis in osteosarcoma. Therefore, curcumin has many great properties that play important roles in osteosarcoma treatment. Naboneeta and Susmita reported that an implant based on curcumin loaded hydroxyapatite-coated titanium improved MG-63 cytotoxicity in vitro [61]. Another study by his group suggested that curcumin incorporated in a 3D-printed calcium phosphate scaffold showed selective toxicity toward MG-63 cells and supported the normal proliferation of osteoblasts [62]. Another asset of this combination approach is the increased accumulation of curcumin in the damaged tissue area. Because of some curcumin disadvantages such as extensive first pass metabolism and poor bioavailability [63], conventional delivery strategies cannot overcome these problems. By incorporating curcumin in such materials, it can properly accumulate in the target area, resulting in a pharmacological potency boost [64].

Table 2 contains the in vitro/in vivo effects of various polyphenols against different cancer types.

## 4. Combination of Polyphenols with Other Agents to Enhance the Therapeutic Outcome

Polyphenols can act synergistically when mixed with clinically used anticancer agents. Their improved anticancer activity at lower chemotherapy-associated toxicity on normal tissues could be due to their improved pharmacodynamics, pharmacokinetics, bioavailability, and metabolism. Considering the encouraging preclinical and clinical utilization, more attention has been paid to the design of drug loaded nanoparticles, liposomes, nanocarriers, and micelles to increase the drug efficacy and target specificity [4].

There are two major issues associated with cancer chemotherapy: frequent adverse side effects and multidrug resistance. Lately, chemotherapy has slowly passed from a mono-substance to multidrug therapy. Therefore, the drug cocktail approach has become more widely known and used. For instance, tea is a commonly consumed beverage by many people including cancer patients due to its health benefits. Catechins, a class of polyphenols, are the most important bioactive molecules found in green tea. The mixture of green tea catechins and classical anticancer drugs has started to be of interest in cancer treatment. Studies have reported that this type of combination could improve the treatment outcome and reduce the side effects of classical antitumorals in cancer patients [77]. 

Bleomycin is a chemotherapeutic agent commonly used to treat different cancers. Although the monotherapy approach has repeatedly failed to generate the desired therapeutic outcomes because of the development of drug resistance. Therefore, tea polyphenols have been included as adjuvants in bleomycin therapy. Alshatwi et al. [78] showed that a combination of bleomycin and tea polyphenols had a synergistic anticancer activity. The study consisted of treating cervical cancer cells (SiHa) with various concentrations of bleomycin, tea polyphenols, and a combination of both. The effects related to cell growth, reactive oxygen species level, early apoptosis, poly-caspase activity, and the expression of BCL-2, caspase-8, caspase-9, and p53 were investigated. The results suggest that this combination acts synergistically and reduces the cervical cancer cell viability and proliferation through apoptosis. Other research has pointed out that tea polyphenols enhance bleomycin antitumor activity [77].

Cisplatin is one of the most frequently used anticancer drugs. It is generally considered as a cytotoxic agent that destroys cancer cells through the formation of inter- and intra-strand DNA crosslinks and cisplatin DNA adducts. The mode of action is based on complex molecular mechanisms that include multiple events that involve inflammation through pro-inflammatory factor activation, ROS production, and lipid peroxidation, and the stimulation of p53-dependent signaling pathways. Although cisplatin use has been reported to lead to severe side effects such as ototoxic damage and this was why, in a first trial, if possible, loco-regional delivery is exploited [79], but nowadays, more often, combination therapy with natural agents is recommended. Elevation of the hearing threshold was registered, especially for higher frequencies, in 22 to 75% of adults treated with cisplatin and 26 to 90% of young patients. This led to severe and permanent hearing loss, which is a debilitating process. More and more researchers have focused on trying to explain the molecular mechanisms leading to cisplatin resistance and toxicity including inflammation and oxidative stress. Nevertheless, these molecular mechanisms are still poorly understood [80].

Fabiola et al. evaluated the effects of two polyphenols, ferulic acid and curcumin, used as adjuvant agents both in vitro and in vivo. In vitro, they studied the transcription factors that play a role in tumor progression and cisplatin resistance and in vivo, they investigated their antioxidant ability to protect against cisplatin ototoxicity. It has been shown that both curcumin and ferulic acid have antioxidant and otoprotective effects on the cochlea through the downregulation of p53 phosphorylation and Nrf-2/HO-1 upregulation. Although curcumin is only able to act on inflammatory pathways involved in the inhibition of NF-κB activation, one great aspect is that curcumin converts antioxidant effects into pro-oxidative and anti-inflammatory effects in human cancer cells. Curcumin possesses chemosensitive properties through targeting some cisplatin chemoresistant factors such as NF-κB, Nrt-2 and Stat-3 phosphorylation. Ferulic acid has a biphasic response: it has pro-oxidative effects at lower concentrations and antioxidant effects at higher concentrations, stimulating chemoresistance. Therefore, polyphenols, especially curcumin, hold a great promise in cancer therapy. Due to this biphasic response, meaning the ability to act as both an antioxidant on normal cells and pro-oxidant on tumor cells, these natural compounds probably involve an interaction between the key factors NF-κB, Nrt-2, Stat-3, and p53 [80]. 

The combination of cisplatin and tea polyphenols can reduce and slow down the appearance of drug resistance, which could also minimize the drug toxicity and improve the therapeutic outcome. The mixture of cisplatin and EGCG improved the cell cycle arrest and antioxidant activity compared to monotherapy and regulated apoptosis and ROS-related gene expression. Tea polyphenols could also stop the proliferation and chemosensistization of ovarian cancer cells to cisplatin. This combination can stop various cancer cells growth including MCF-7 breast cancer cells and A549 lung cancer cells [77].

Ibuprofen is a medication in the nonsteroidal anti-inflammatory drug (NSAID) class that may be able to reduce the growth of prostate cancer cells in both in vitro and in vivo xenograft models. The combination between EGCG and ibuprofen has been tested on DU-145 prostate cancer cells and revealed that the mixt treatment led to better growth inhibition than both substances alone. This combination can stop proliferation and stimulate apoptosis in DU-145 prostate cancer cells [77,81]. 

Tamoxifen is a selective estrogen receptor modulator used to prevent breast cancer in women and treat breast cancer in women and men. Green tea is usually taken as a supplement in the prevention and treatment of breast cancer. It has been reported that the synergistic use of tamoxifen and green tea leads to better therapeutic outcomes in breast cancer cell lines and experimental animal models. Green tea acted as an adjuvant, enhancing the tamoxifen effects on the proliferation of estrogen receptor-positive ZR75, MCF-7, and T47D human breast cancer cells in vitro. MCF-7 xenograft-bearing mice were treated with a combination of tamoxifen and green tea and showed a decreased tumor size and increased apoptosis of cancer cells in the affected tissue [77].

Bortezomib is an anticancer medication mainly used to treat multiple myeloma and mantle cell lymphoma. It acts by reversibly blocking the 26S proteasome. It has been observed that EGCG affects bortezomib anticancer activity by lowering its efficacy in a concentration dependent manner when tested on CWR22 xenograft-bearing breast cancer mice. Low levels of EGCG did not affect the CWR22 mice, while very high concentrations antagonized the anticancer activity of bortezomib. This suggests the fact that EGCG could also have a negative effect when combined with an anticancer drug [77].

The potentiating effect of the anticancer activity of tea polyphenols is also reflected in other antitumor drugs such as sulindac, docetaxel, paclitaxel, retinoids etc. Studies showed that a daily dose of green tea extract did not affect the cytotoxicity of 5-fluorouracil treatment in the BGC823, SW480, and BIU-97 human cancer cell lines [77,82].

The combination of apigenin, rhein, quercetin, and emodin with cyclophosphamide and cisplatin promoted cell cycle arrest, apoptosis, and DNA damage and reduced the glutathione and ATP levels in the lymphoid leukemia cell lines. The combination of polyphenols with alkylating agents, especially cisplatin, seems to be a promising tool in lymphoid leukemia treatment, with apigenin having the strongest effects. Additionally, in myeloid cells, apigenin showed the same beneficial effects when mixed with alkylating agents, suggesting that it could also be used in myeloid leukemia [83].

Another study investigated the synergistic use of paclitaxel and apigenin in ovarian cancer. A549, HEK293A, Hep3B, and HeLa cells were treated with a combination of paclitaxel and apigenin, showing that both compounds improved apoptosis through the reduction in the number of surviving cells. Apigenin can also be used together with cisplatin. For instance, HK-2 cells (human renal proximal tubular epithelial cells) treated with this mixture presented a reduction in p53 activation and subsequent stimulation of the PI3K/Akt pathway. In PC-3 cells of prostate cancer and cancer stem cells, the synergistic effect led to the suppression of PI3K/Akt activation and NF-κB protein expression. Moreover, apigenin combined with doxorubicin promoted a stronger reduction in the ATP levels in the Jurkat, CCRF-CEM, and THP-1 leukemia cell lines. Therefore, this combination decreased the ATP levels in three out of four leukemia cell lines by improving the toxicity and DNA damage. It also enhanced the caspase-3 activity and cell cycle arrest in all four cell lines [84].

Primary colon cancer is usually treated with dasatinib, a drug that inhibits the SRC-family of protein kinases. However, sustained administration of this chemotherapeutic compound leads to drug resistance and tumor development. In order to diminish these negative effects, dasatinib was mixed with curcumin and tested in both in vitro and in vivo. The results showed an increased inhibition in numerous metastatic processes. When tested in vitro, curcumin and dasatinib improved the cell adhesion phenotype of the HCT-116 colon cancer cells. Additionally, in the APCMin+/− mice, the mixture led to the regression of intestinal adenomas of 95%, reducing tumor progression and improving apoptosis [84,85].

Another combination of pterostilbene and EGCG was evaluated on the MIA PaCa-2 and PANC-1 pancreatic cell lines. This combination induced cell cycle arrest in the S-phase in the case of MIA PaCa-2 cells, but not in the PANC-1 cells. It also stimulated mitochondria depolarization and cytochrome-C upregulation in the MIA PaCa-2 cells, but not in the PANC-1 cells. An increased apoptosis in PANC-1 was observed in contact with the MIA PaCa-2 cells. Thus, enhanced antitumoral effects are achieved when combining pterostilbene with EGCG [84,86].

The complex aspects of the antitumoral benefits of EGCG combined with ascorbic acid, copper, selenium, manganese, lysine, arginine, proline, and N-acetylcysteine on various cancer cell lines have been studied in vitro and in vivo. This combination showed a great potential in reducing the pulmonary and hepatic metastasis in vivo, leading to an important decrease in the tumor size in all of the tested human cancer cell lines. In vitro tests showed that EGCG-manganese can effectively suppress cell proliferation, cell invasion, cell migration, angiogenesis, stimulated apoptosis, and pro-apoptotic genes in various cancer cell lines [38].

Doxorubicin is a very commonly used antineoplastic drug that has had efficient results in treating hematologic cancers and malignant neoplasms in both children and adults, although its use has become quite problematic because of the appearance of acute and chronic cardiotoxicity, causing severe cardiomyopathy and congestive heart failure. Even though the complete molecular pathogenesis of anthracycline cardiotoxicity has not been fully determined, some studies have demonstrated that the main cause is the intramyocardial production of ROS. Considering this hypothesis, the development of protective approaches using natural antioxidants that do not affect doxorubicin efficacy is certainly needed [87].

Various studies have shown that the heart possesses much lower amounts of enzymatic defenses against the harmful effects of free radicals in contrast to other organs and suffers for a long time during the cardiac detoxifying process after being exposed to doxorubicin anthracycline. Additionally, it has been proven that cardiomyocytes do not possess the proper mechanisms to remove hydroperoxide generation by their own enzymatic defenses. One interesting mechanism that makes doxorubicin promote ROS formation is its high affinity to iron. When doxorubicin interacts with iron, it suffers a reduction reaction, inducing a redox cycle of ROS generation, and the emerging metabolite doxorubicinol further interacts with the thiol groups on proteins, leading to more cell damage [87].

It has been reported that the dietary administration of antioxidants from plants, together with cytostatic drugs, is able to reduce chemotherapy-related cardiotoxicity and the probability of developing cardiomyopathy and heart failure. This ability has been observed for some vitamins such as C and E, which, when used separately or mixed, can avoid the detrimental effects of lipid peroxidation without decreasing the antitumoral properties of doxorubicin. Sin et al. showed that resveratrol has the ability to protect against doxorubicin-caused cardiotoxic side effects in a SAMP8 model (old senescence-accelerated mice). Resveratrol inhibited doxorubicin-induced deficiency of cardiac systolic function by reestablishing SIRT-1 activity to diminish USO-7-related catabolic/pro-apoptotic signaling in aged animals [88]. Some other studies have demonstrated that quercetin is able to reduce the levels of lipid hydroperoxide by cancelling ROS via two ways: by directly reacting with NO, O_2_, and ONOO or by removing peroxyl radicals and alkoxyl lipid. Moreover, due to its antioxidant capacity, quercetin has several other important effects. When mixed with losartan, it can enhance the protective effects against chronic doxorubicin-induced cardiotoxicity. Quercetin also showed another important benefit on cardiac protection when co-administered with losartan by reducing the levels of pro-inflammatory cytokines and strongly reestablishing the normal antioxidant enzyme activity [87].

Curcumin was also administrated together with some conventional antitumoral drugs to potentiate their anticancer activity. In different cancer cells, the co-administration of curcumin and cisplatin showed improved anticancer activity, especially by blocking the phosphorylation of the ERK signaling pathway. When mixed with oxaliplatin, curcumin showed great inhibition of oxaliplatin-resistant colorectal cancer cell proliferation via the regulation of the NF-kβ signaling pathway [89]. Curcumin co-administered with doxorubicin showed an induction of chemo-sensitization by cell cycle arrest and the stimulation of apoptosis. Moreover, curcumin stimulated the accumulation of doxorubicine inside the cells by downregulating P-glycoprotein, which improved the anticancer efficacy. Curcumin, together with paclitaxel, modulated the paclitaxel uptake via the modulation of folate receptors in breast cancer cells. Curcumin enhanced the paclitaxel-mediated modulation of the MMP-9 and NF-kβ signaling pathway aside from ROS production to stimulate apoptotic cell death. Additionally, curcumin co-administered with paclitaxel stimulated caspase-mediated apoptosis in human HeLa cervical cancer cells. This combination was also tested on Hep3B and HepG2 hepatoma cells and demonstrated a synergistic action via downregulation of Lin28. In human bladder Ku-7 cancer cells, the co-administration of curcumin and paclitaxel led to the regulation of NF-kβ signaling and reduction in angiogenesis. Curcumin, together with red and blue light irradiation, led to oxidative stress and ER-stress mediated apoptosis in A375 melanoma cells. Curcumin has been used in combination with celecoxib and gemcitabine and has reached phase II and III clinical trials in pancreatic cancer. Curcumin, in combination with capecitabine and radiotherapy, has also entered into phase II clinical trials, which was used in rectal cancer [4].

The beneficial effects of curcumin when co-administered with doxorubicin on cancer suppression were carefully analyzed by investigating the induced DNA fragmentation in tumor cells by the TUNEL assay and then studying programmed apoptotic cell death by Annexin, which is an early apoptosis biomarker. The results showed that the combination therapy had great anti-cancer effects without affecting cardiac health, even at lower doxorubicin concentrations, so doxorubicin toxicity can be avoided. Curcumin can also regulate gene expression involved in inflammatory responses and apoptotic signaling, which characterizes cancer cells upon chemotherapy or combined therapy (cytostatic and natural compounds). The advantage of curcumin use is related to a serious reduction in anti-apoptotic biomarker expression, which leads to increased cell death. It also strongly enhances pro-apoptotic and anti-inflammatory responses, therefore stimulating cancer cell death [90].

As previously mentioned, EGCG and its combinations have been stated as great anticancer compounds that are able to induce apoptosis. EGCG used either alone or combined with various natural molecules have been reported to stimulate the apoptosis process in many cancer types. The hypothesis is that by mixing two or more therapeutic compounds, more pathways could be targeted, which will lead to an enhanced drug stability and lower toxicity, so that better therapeutic outcomes can be expected. Numerous findings have shown that EGCG can act in a synergistic manner by stimulating cancer cell apoptosis both in vitro and in vivo when combined with various natural molecules such as curcumin, vitexin-2-*O*-xyloside and raphasatin, pterostilbene, *N*-acetylcystine, quercetin, grape seed extract, 5-fluorouracil, etc. It is involved in the upregulation of pro-apoptotic proteins and the downregulation of anti-apoptotic proteins [27]. 

Resveratrol has also been reported as a great enhancer of the anticancer potential of classical antitumor drugs in numerous cancer types. For instance, when tested on a malignant glioma xenograft mouse model, resveratrol was combined with temozolomide and presented high anticancer efficacy. It seems that the mechanism behind this is that the combined use leads to apoptosis via the inhibition of ROS/ERK-mediated autophagy. Additionally, resveratrol co-administered with paclitaxel showed a greater anticancer potential in breast cancer than the single administration of each drug [89]. In B16 melanoma bearing mice, the use of resveratrol, together with doxorubicin, diminished the resistance acquired against doxorubicin. Moreover, it affected the cell cycle and induced apoptosis, thereby increasing the survival rate of tumor-bearing models [4,91].

Resveratrol co-administered with 5-fluorouracil stimulated S-phase cell cycle arrest in murine hepato-carcinoma. When combined with rapamycin, which is an autophagy inducer of mTORC1, it showed an increased anticancer efficacy both in vitro and in vivo. This combination induced autophagy in breast cancer cell lines by decreasing Akt phosphorylation. In MSTO-211H cells (human malignant mesothelioma), the co-administration of clofarabine and resveratrol improved cancer spread by regulating the PI3K/Akt/mTOR signaling pathway [4]. 

Adahoun et al. investigated the improvement in the bioavailability and solubility of curcumin nanoparticles obtained through a method focused on a wet-milling technique. These were tested in vitro against the PC3-prostate cancer cell line, human erythrocytes, HEK (human embryonic kidney) cell line, and on four bacterial strains: two Gram-negative (Pseudomonas aeruginosa ATCC 27853 and Escherichia coli ATCC 25922), and two Gram-positive (Staphylococcus aureus ATCC 29213 and Micrococcus luteus ATCC 9241). The attention was focused on the cell viability profile, the minimum bactericidal concentration, and the half maximal inhibitory concentration. Nanocurcumin showed great effects against PC3 and reduced toxicity against HEK healthy cells when compared to typical curcumin in favor of PC3 (P50.05). Moreover, it was determined that nanocurcumin displayed much higher toxicity against PC3 than HEK compared to typical curcumin. The obtained data report that even though nanocurcumin is somehow more liable to lay red blood cells than typical curcumin after 60 min, the hemolysis stayed very low and there was no important difference between nanocurcumin and typical curcumin induced hemolysis. However, the minimum bactericidal concentration was lower for nanocurcumin than curcumin for all four bacterial strains. Additionally, Gram-positive bacteria were more sensitive to both nanocurcumin and typical curcumin when compared to Gram-negative bacteria. Therefore, these findings suggest that nano-scaled curcumin has greater advantages and significant ability to treat prostate cancer and infections [92]. 

Curcumin has been reported to reduce the gastrointestinal toxicity associated with chemotherapy. For instance, in the case of 5-FU-induced, it reduced the gastrointestinal toxicity by increasing the intestinal permeability and inhibiting mucosal damage. Moreover, curcumin administration reduced gastrointestinal toxicity induced by methotrexate by improving intestinal permeability. Curcumin proved to be beneficial against doxorubicin-related cardiotoxicity by regulating calcium flux alteration, oxidative stress, mitochondrial damage, and the installation of the apoptotic process in cardiac tissue. In those cells treated with curcumin, it has been observed that curcumin is able to downregulate the expression of SCK (the cardiotoxic marker) and to upregulate SOD (superoxide dismutase) and CAT (catalase) expression [93]. When co-administered with cisplatin, curcumin improved the expression of SOD and CAT, which are especially involved in hepatoprotection. When used together with doxorubicin, curcumin balanced the ALT (serum alanine aminotransferase) and AST (aspartate aminotransferase) levels in order to assure hepatoprotection. It also reduced cisplatin and oxaliplatin-induced hepatotoxicity by decreasing the glutathione levels and lipid peroxidation and by increasing the mitochondrial respiratory chain enzymes to assure hepatoprotection. Regarding methotrexate hepatotoxicity, the administration of curcumin reduced nephrotoxicity by decreasing the expression of creatinine and blood urea nitrogen [4,94]. 

Resveratrol has also been assessed to inhibit cardiotoxicity caused by doxorubicin and daunorubicin. It reduced doxorubicin-mediated dilated cardiomiopathy, myocarditis, and congestive heart failure during cancer treatment. Doxorubicin leads to apoptotic cell death caused by ROS in cardiac tissue [95]. The use of resveratrol, which is a strong antioxidant, is able to clean ROS in order to prevent the apoptosis of cardiac tissue. It raises superoxide dismutase activity, which is the main enzyme able to remove doxorubicin-mediated ROS generation. Doxorubicin is responsible for calcium regulation disruption in cardiac tissues and further modulates aldo-keto reductase and carbonyl reductase enzymes that are involved in cardiac toxicity. The use of resveratrol blocks these two enzymes, which offers cardiac protection in the case of doxorubicin treatment. Resveratrol improved the anticancer activity and diminished arsenic trioxide cardiotoxicity in hematological cancers via the removal of oxidative stress by using its antioxidant potential. Additionally, in arsenic trioxide treatment for leukemia and multiple myeloma, resveratrol was able to reduce nephrotoxicity and hepatotoxicity by diminishing arsenic accumulation in the liver. Resveratrol modulated several key molecules involved in cell cycle and apoptosis to offer cryoprotection from photocarcinogenesis [4]. 

Xiang et al. developed a new polyphenol–metal coordination strategy to manufacture cisplatin nano-formulations. The particles of PEG-GAx/Pt were characterized by increased and tunable drug loading contents and have a high stability in physiological conditions. Cisplatin is released according to the acidic pH and ROS levels. They are also characterized by prolonged blood circulation times and improved tumor accumulation, leading to better anticancer activity and lower toxicity [96].

Jiening Dun et al. studied the therapeutic activity and anticancer mechanisms for the co-administration of resveratrol and 5-fluorouracil. This combination was tested on the TE-1 and A431 cancer cell lines and an animal model for melanoma. It seems that resveratrol is able to potentiate 5-fluorouracil effects and induce S-phase arrest. These substances act synergistically, leading to tumor regression when tested in a two-model of mouse skin cancer. Resveratrol increased growth inhibitory effect of 5-fluorouracil in vitro on A431 and TE-1 cancer cells. When tested in vivo, it was discovered that this combination led to an important tumor regression rate after four weeks of treatment. Resveratrol and 5-fluorouracil strongly enhanced the percentage of apoptotic cells and the caspase-3, p53, and cleaved PARP levels while also raising the BAX/BCL-2 ratio. Therefore, the use of a combination of resveratrol and 5-fluorouracil treatment is more effective in blocking cancer cell growth and the induction of apoptosis [97].

Wang et al. demonstrated that the addition of an anti-inflammatory lignan extracted from Arctium lappa seeds, known as arctigenin (Arc) to EGCG and curcumin, enhanced the chemopreventive potential in the MCF-7 breast cancer and LNCaP prostate cancer cell lines, when compared to the treatment with each substance alone. Both EGCG and Arc improved the apoptotic effects of curcumin in the LNCaP cells. The same effect was obtained in the MCF-7 breast cancer cells by the Arc and curcumin combination. The synergistic effect manifested through the inhibition of NF-κB, PI3K/Akt, and STAT3 expression underly the potential benefits in clinical practice [35,98].

Co-administration of EGCG and doxorubicin showed a great ability to induce liver cancer cell death. Chen et al. pointed out that EGCG had the ability to inhibit autophagic activity and stopped hepatoma Hep3B cell proliferation both in vitro and in vivo depending on the time and dose [35,99,100].

Table 3 contains the in vitro/in vivo effects of multidrug therapy on various cancer types.

## 5. Polyphenol-Based Biomaterials for Cancer Treatment

Drug delivery systems based on natural polyphenols have received a lot of attention in the last years. An important advantage of polyphenols is that they can be oxidized into nanoparticles, oligomers, and polymers under mild and physiological conditions. The resulting materials could be used in many applications such as photothermal therapy, drug delivery, antibacterials, and sunscreen. Additionally, natural polyphenols that have various catechol/pyrogallol groups are relatively easily conjugated with different polymers in order to obtain functional materials [101].

The catechol and pyrogallol groups found in natural polyphenols can effectively complex with a wide number of metal ions to obtain interesting metal–phenolic coatings. For instance, nanoparticles obtained from doxorubicin loaded calcium carbonate and a metal-organic framework were coated with Fe(III)/EGCG or Al(III)/tannic acid metal-phenolic capsules to be used in targeted drug delivery. At the same time, self-assembled drug nanoparticles based on carfilzomib and paclitaxel were coated with Fe(III)/tannic acid for sustained drug release. Fe(III)/tannic acid coated nanoparticles can additionally be functionalized with folate-poly(ethylene glycol) for targeted cancer therapy [101].

Hydrogels are a type of material frequently used in drug delivery. The catechol and/or pyrogallol groups found in natural polyphenols typically allow for crosslinking with several other species through covalent bonding, for instance, obtaining diester bonds with boronates and coordination with metallic ions. Tannic acid is a natural polyphenol that has great gelation properties and is used to fabricate hydrogels due to its exceptional solubility and the increased density of pyrogallol and catechol moieties present on the surface. Tannic acid is able to create stable hydrogels with Ti(IV) or Zr(IV) ions [102]. Even though Fe(III) is characterized by the highest coordination constant with tannic acid, it has been observed that it can form a gel in combination with tannic acid. Ti(IV) has a high oxidation state that is efficient for solvent trapping during gelation. Tannic acid/Ti(IV) gels can encapsulate bioactive compounds to manage their crystallization and release behavior [101,103].

Gene carriers based on polymers typically bind nucleic acids through ionic interactions, therefore, high molecular weight polymers with a high density of positive charges are needed to properly condense these molecules into nanoparticles, although high molecular weight polymers are toxic for treated cells. On the other hand, low molecular weight polymers that have less positive charges are not able to properly bind nucleic acids and cannot bring these molecules into cytosols. To overcome this issue, natural polyphenols with increased binding affinity with DNA and siRNA were used to stimulate the condensation of nucleic acids by low molecular weight cationic polymers. For instance, siRNA was combined with EGCG and created uniform negatively charged nanoparticles that can be coated with cationic polymers to obtain core-shell nanoparticles [104]. EGCG significantly promoted siRNA molecules, binding to low molecular weight polymers and allowed for great and nontoxic gene delivery. Due to this promising result, more polyphenols such as catechols have been linked to low molecular weight polymers to fabricate polycatechols for siRNA delivery [101,105]. 

Numerous studies have suggested that the anti-cancer activity of singular agents could be significantly improved when mixing them with various compounds that are chemically similar, making them act synergistically. This kind of combination could act potently to decrease drug dosage and resistance while also presenting a better therapeutic outcome. It has been demonstrated that EGCG, in combination with several dietary agents (curcumin, quercetin, [6]-gingerol, lovastatin, panaxadiol, sulforaphane, and pterostilbene), is able to act in a synergistic manner and suppress cancer cell proliferation in vitro and in vivo. EGCG also attained great results when used in combination with chemotherapeutic drugs such as capecitabine, 5-fluorouracil, cisplatin, temozolomide, docetaxel, and doxorubicin, or several other compounds (amino acids, vitamin C, and sodium butyrate). These EGCE combinations are able to synergistically induce apoptosis, suppress cancer cell proliferation, and inhibit tumor angiogenesis and progression. This significant advantage can be related to improved EGCG bioavailability [27].

New fluorescent core shell polymeric nanoparticles based on biodegradable materials were obtained by Asadi et al. to act as a carrier for the prolonged, targeted, and controlled release of 5-fluorouracil. The cross-linker agent was obtained using tannic acid, which is a biodegradable polyphenol with great anti-cancer potential. Furthermore, the mini-emulsion polymerization method was used together with magnetic florescent cores in order to develop the carrier. Nontoxicity of the samples was stated by the MTT viability assay. When drug release was tested in vitro, it showed a faster profile in the simulated gastric fluid than the physical conditions. These carriers showed improved anti-cancer properties when tested on human breast cancer cells [106].

A mixture of paclitaxel and curcumin was loaded into albumin nanoparticles for the treatment of SKOV-3 ovarian and HeLa cervical cancer cells. The albumin nanoparticles were able to stimulate apoptosis in these cancer cells. In a similar way, mesoporous silica nanoparticles loaded with both curcumin and paclitaxel suggested a potent anticancer activity with enhanced pharmacokinetics [107]. Additionally, silica nanoparticles penetrated effectively into sub-cellular organelles (i.e., lysosomes, mitochondria), suggesting a potential activation of mitophagy and autophagy. Curcumin-loaded nanoparticles involved in the modulation of P-glycoprotein activity were able to sensitize cancer cells toward paclitaxel. They had better results when tested on ovarian cancer cells because of the dynamic modulation of CD44. Even better results were obtained by RGD-associated nano-liposomes loaded with either curcumin alone or a combination of curcumin and paclitaxel. These nano-liposomes suppressed angiogenesis and metastasis in A549 lung cancer cells through integrin binding. In KYSE-30 human esophageal squamous cancer cells, micelle based drug delivery systems loaded with curcumin showed important results in removing the drug resistance and stimulation of cell cycle arrest by downregulating the expression of Cyclin D. Additionally, curcumin and paclitaxel loaded in micelle vehicles were more effective against A549 cells by reducing the drug resistance and improving chemo-sensitization [4,108]. 

Resveratrol loaded in polymeric nanoparticles showed great efficacy against LNCaP prostate cancer cells through the regulation of ROS-mediated apoptosis. Moreover, they supported G1-S phase cell cycle arrest and suppressed cell proliferation. Additionally, solid lipid nanoparticles loaded with resveratrol had significant anti-proliferative effects against MDA-MB-231 breast cancer cells. They stimulated intrinsic apoptotic cell death through the downregulation of Bcl-2 and upregulation of Bax. Furthermore, the suppression of c-Myc and cyclin D1 expression led to anti-proliferative effects. Likewise, gold nanoparticles functionalized with resveratrol proved to be effective against PC-3 prostate cancer, MDBA-MB-231 breast cancer, and PANC-1 pancreatic cancer cells. Resveratrol encapsulated in gelatin nanoparticles showed an improved anti-cancer activity against NCI-H460 non-small cell lung carcinoma cells. They promoted oxidative stress, causing DNA damage and apoptotic cell death [4,109]. 

Polymeric nanoparticles can be obtained in the form of nanocapsules or nanospheres from a variety of natural, synthetic, and semi-synthetic polymers. Nanospheres are represented by a continuous matrix, which can encapsulate therapeutic agents through the use of reverse micelles, pH modification, or the incorporation of polyanion. It has been observed that a higher encapsulation efficacy is obtained when the therapeutic agent is molecularly dispersed in the polymeric matrix, and then adsorbed onto the surface. Nanocapsules are represented by a liquid core, which can be either oil or water, and can encapsulate the drug and a polymeric shell. The shell is a thin polymeric layer that protects the therapeutic agent found inside the core and controls the release profile. The required polymer quantity is more reduced in the case of nanocapsules. Considering curcumin’s very low water solubility, it has been incorporated into nanoparticles in order to enhance its therapeutic effects and bioavailability. Poly-L-lactic-co-glycolic acid (PLGA) nanoparticles were used to encapsulate curcumin and then tested on the Hep-2 human laryngeal squamous carcinoma cell line to investigate the influence on cell proliferation and the expression of the caspase-3, apoptotic biomarker. It has been determined that there is an increased cytotoxic effect and higher caspase-3 expression in the case of cells treated with curcumin-loaded PLGA nanoparticles. Apoptosis inducing capacity is time dependent because curcumin nanoparticles seem to be more efficient in short-term exposure. The results were argued by the different cellular uptake in correlation with the contact time of nanoparticles with cells [110]. Despite being an excellent anti-proliferative and chemopreventive agent, resveratrol is highly photosensitive and has low chemical stability. By being loaded into PLGA nanoparticles, it assures a strong protection against degradation caused by light exposure. Resveratrol has also been encapsulated into various polycaprolactone (PCL) nanoparticles, and skin penetration profiles have been determined by testing them on porcine skin mounted in vertical diffusion cells, followed by tape stripping and the differentiation of the viable epidermis and dermis. The results showed that photo-isomerization was highly delayed without being influenced by the nanoparticle type. Luteolin is also a natural polyphenol with antioxidant properties that is found in a glycosylated form in some green vegetables. It is a great anti-inflammatory and anticancer agent. Antitumoral effects have been observed in a number of cancers including the skin. Because it is hydrophobic and has low bioavailability and systemic delivery, luteolin has been loaded into micelles made of monomethoxy poly(ethylene glycol)-poly(epsilon-caprolactone) (OMe-PEG-PCL). The anticancer effects were evaluated against Tu212 squamous cell carcinoma of the head and neck cells. An IC50 value of the functionalized nanoparticles was 4.13 μmol/L compared to 6.96 μmol/L recorded for the free luteolin underlining the chemopreventive effects of nanoparticles. Another great therapeutic agent, gallic acid, was encapsulated in the PLGA nanoparticles coated or not with Tween 80 and the obtained formulation’s cytotoxicity was tested on red blood cells. The results showed that gallic acid did not induce hemolysis and exhibited significant antioxidant effects for the uncoated nanoparticles [111,112].

Bioactive glasses have been intensively investigated as biomaterials for bone tissue applications. M. Cazzola et al. studied the possibility of functionalizing the surface of bioactive glasses with plant polyphenols (for instance, gallic acid and polyphenols extracted from green tea leaves and red grape skin) in order to stimulate the anti-cancer activity in vitro. The performed assays were direct and indirect cytotoxicity on the U2OS bone osteosarcoma cells and hFOB human fetal pre-osteoblasts, together with the quantification of nitrogen and oxygen species produced by cells as a response to the material. Then, the DNA damage of the U2OS cells after coming in contact with bioactive glass was assessed to analyze any selective cytotoxic effect of the modified materials against tumor cells. Additionally, the response of the hFOB healthy and U2OS cancerous osteoblast cells to the polyphenol functionalized and un-functionalized bioactive glass was illustrated by the direct assay against U2OS. Tea polyphenol functionalized bioactive glass showed great potential to stimulate reactive oxygen and nitrogen species production, and to also offer a kind of anti-inflammatory protection for hFOB. Eventually, irreparable DNA damage, along with some sort of difficulty in generating tumor aggregates, was revealed for U2OS cancer cells cultured on a bioactive glass functionalized with tea polyphenols. These findings show that polyphenol functionalized bioactive glasses may be very promising biomaterials for bone substitution in cancer treatment [113]. 

In a study by Vasconcelos et al., some resveratrol based emulsifying drug delivery systems were designed and revealed lower metabolism, enhanced solubility, and increased pharmacokinetic profile [114]. In a study by Vasconcelos et al., some resveratrol based emulsifying drug delivery systems were designed and revealed lower metabolism, enhanced solubility, and increased pharmacokinetic profile. Some nanoparticles based on resveratrol-loaded glycyrrhizic acid-conjugated human serum albumin were evaluated in vivo on rats by single dose injection in the tail and reported enhanced bioavailability and increased concentrations found in the main organs when compared to simple resveratrol. The most quantitatively important concentrations were found in the liver, showing that it could represent a promising liver-targeted delivery system [115]. 

Resveratrol-bovine serum albumin nanoparticles ware obtained by Guo et al. and showed increased dispersal and water solubility. These were able to suppress cancer cell growth in nude mice holding human primary ovarian tumors [116].

Another study by Ciobanu et al. studied the effects of a novel bio(hybrid) matrix based on mesoporous silica nanoparticles loaded with *Althaea officinalis* and *Betonica officinalis* extracts. The evaluation of the two plant extracts’ cytotoxicity against the Hep-2 cancer cell line underline the fact that the *Althaeae folium* extract can inhibit Hep-2 cancer cell viability by decreasing the access of cancer cell to nutrients from the media, while the *Betonica officinalis* extract was able to inhibit the Hep-2 cancer cell viability by its interaction with the inner metabolism of the cell. Considering this, novel great anticancer biocomposites are expected when mixing the *Althaeae* and *Betonica* extracts [117].

Gallic acid (GA) functionalized chitosan (CS-GA) and caseinophosphopetides (CPP) were combined to obtain the CS-GA-CPP nanoparticle by the self-assembly method. GA offers antioxidant and anticancer properties. Additionally, EGCG was loaded into CS-GA-CPP nanoparticles during the self-assembly, with more than 80% loading efficiency. Nanoparticles are able to protect EGCG, which is unstable in alkaline and neutral conditions, from oxidative processes during delivery. The controlled release of EGCG can be easily obtained in the simulated gastrointestinal media and the release of EGCG presented anticancer effects against colon cancer cells. Thus, these nanoparticles represent a great alternative for the oral administration of bioactive polyphenols for the treatment of colon cancer and digestive tract diseases [118,119].

Dziadek et al. fabricated multifunctional PCL/bioactive glass composites functionalized with polyphenols from sage by the solvent-casting method [120]. Dziadek et al. fabricated multifunctional PCL/bioactive glass composites functionalized with polyphenols from sage by the solvent-casting method. These materials presented enhanced hydrophilicity and great in vitro bioactivity. Additionally, they had significant antioxidant and antiproliferative effects against WM266-4 cancer cells. These results suggest that these composites are promising biomaterials for bone tissue regeneration after resection. Additionally, this strategy can be used for the production of 3D scaffolds.

## 6. Limitation of Polyphenols as Anticancer Agents

Plant-derived polyphenol compounds have been intensively studied for cancer therapy because they are able to modulate apoptotic genes. However, there are some factors that limit the use of phenolic compounds in humans: low solubility, low bioavailability, poor permeability, rapid release, instability, and susceptibility to environmental factors [121,122].

Polyphenols have a less than optimal pharmacokinetic profile. Some polyphenols have very low efficiency because of their low bioavailability, the consumed quantity, and the type and stage of cancer. Thus, the variable response to polyphenols is influenced by dose, the cancer cell type, and the patient’s genome [123].

As previously mentioned, one of the greatest challenges associated with the therapeutic use of polyphenols is their low oral bioavailability. The absorption, transportation, bioavailability, and bioactivity of polyphenols are very important for their efficacy as new drug candidates. Following oral administration, polyphenols travel through the digestive system, being absorbed in the stomach and small intestine. Some are biotransformed by the gut microbiota or by hepatic phase I/II metabolism, which may affect the bioavailability and bioactivity [124].

It has been suggested that substances with a higher molecular weight did not dissolve effectively in water. Pro-anthocyanidin polymers have substantially reduced water solubility at higher molecular weights, which further results in very poor absorption. Furthermore, phenolic phytochemicals may degrade due to various pH levels in the gastrointestinal tract. For example, EGCG is unstable in an acidic environment (stomach pH below 1.5) and at neutral pH such as in the intestine [125].

Because no particular receptors have been identified to transport phenolic phytochemicals into cells via the surface of the small intestine epithelial cells, polyphenols have minimal intracellular penetration. As a result, paracellular and transcellular diffusions as well as passive diffusion serve as the primary basis for the mechanism of transportation through the epithelium. After being absorbed, phenolic phytochemicals go through an active efflux phase, whereby the majority of them are pushed back into the lumen [125].

Polyphenols are frequently loaded into different carriers to increase their bioavailability, in order to avoid these limitations. Through this process, the biocompatibility will improve, environmental degradation can be avoided, and this could stop other components of the human body from interacting with them. There is evidence that nanocarriers are superior materials for encapsulating phenolic chemicals and increasing their bioavailability [122].

## 7. Conclusions

As cancer cases are rapidly rising and conventional therapies become less effective, new strategies are being evaluated in order to improve the therapeutic outcomes. Considering the various side effects associated with commonly used therapies such as increased toxicity, the development of drug resistance, etc., there has been an increased interest by researchers for the use of natural compounds with antineoplastic properties that are able to protect healthy tissues while destroying tumors without causing additional damage.

Polyphenols have proven to be promising candidates in cancer therapy if associated with chemotherapeutic drugs in order to develop a proper synergy. They have great antioxidant, anti-inflammatory, antiproliferative, antiangiogenic, antimetastatic, and proapoptotic properties. They showed excellent results when tested in various types of cancers including skin, breast, ovarian, colorectal cancers, and osteosarcoma. They inhibited the growth and proliferation of a large number of cell lines and were also able to induce the apoptotic process. Studies showed that polyphenols can act in a synergistic manner when combined with classical antitumorals such as cisplatin, doxorubicin, paclitaxel, 5-fluorouracil, and so on. They can also be loaded into various biomaterials, assuring a controlled release and a combined regenerative and therapeutic action. These findings require further study, especially on in vivo models and in further clinical trials since the mechanism involved in the polyphenol induced regulation of cancer is not completely clear. Additionally, it is important to carefully evaluate the immunomodulatory action of polyphenols to enhance the treatment efficiency such as immune checkpoint blockade. Moreover, prosenescence-polyphenol therapy has gained increased attention because it may minimize toxicity and side effects. However, this strategy should be approached carefully since it can give birth to quiescent tumor cells, leading to cancer recurrence.

## Figures and Tables

**Table 1:** Polyphenols: Properties and sources [8,23].

Polyphenol	Properties	Source	
**EGCG**	Antioxidant Antiproliferative Antiangiogenic Antimetastatic Proapoptotic	Green tea	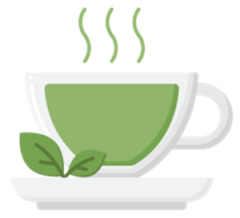
**Curcumin**	Antioxidant Anti-inflammatory Antiproliferative Antiangiogenic Antimetastatic Proapoptotic Chemo and radio sensitizer	Turmeric	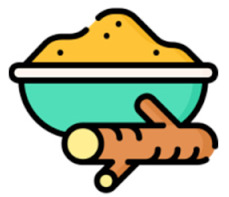
**Caffeic acid**	Antioxidant Anti-inflammatory Antineoplastic Antiviral	Coffee beans	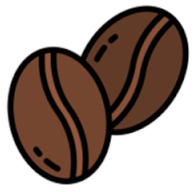
**Resveratrol**	Antioxidant Antiproliferative Antiangiogenic Proapoptotic Chemo and radio sensitizer	Grape	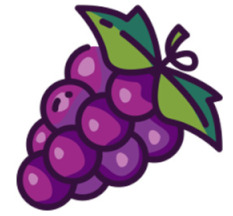

**Table 2:** The effects of polyphenols on various cancers [65,66].

Polyphenol	In Vitro/In Vivo Study	Cancer Type	Dose	Effect	Ref.
Curcumin	In vitro (melanoma cell culture)	Skin cancer	25 µM	Melanoma cell death associated with mPTP opening	[67]
EGCG	In vitro (MCF-7, MDA-MB-231and T47D cells)	Breast cancer	1–40 μM	Inhibiting estrogen-induced cancer cell proliferation, down-regulating ERα, inhibiting metastasis	[68,69]
Apigenin	In vivo (BALB/c-nude mice)	Breast cancer	5–25 mg/kg	Inducing cell cycle arrest through epigenetic change	[70]
Quercetin	In vivo (BALB/c nude mice)	Breast cancer	34 mg/kg	Inhibiting angiogenesis	[71]
Genistein	In vitro (HeLa cells)	Cervical cancer	100 μM	Inducing apoptosis, cell cycle arrest, suppressing cell migration	[72]
Resveratrol	In vitro (PC3 and DU145 cells)	Prostate cancer	25–100 μM	Inducing autophagy-mediated cell death	[73]
Gallic acid	In vitro (HepG2 and SMMC-7721 cells)	Liver cancer	22.1–28.5 μg/mL	Inducing apoptosis	[74]
EGCG	In vitro (HT-29 cells)	Colorectal cancer	1–50 μM	Inducing epigenetic alteration, apoptosis, MAPK and Akt pathways activation	[75]
Resveratrol	In vivo (genetically engineered mouse model for sporadic colorectal cancer)	Colorectal cancer	equal to 105 and 210 mg for human	Suppressing tumor development by modulation of Kras	[76]

**Table 3:** The synergies in cancer multicomponent therapy.

No.	Therapeutically Active Agents	In Vitro/In Vivo Activity	Cancer Type	Effect	Ref.
**1**	Bleomycin and tea polyphenols	In vitro (SiHa cells)	Cervical cancer	Reduced cancer cells viability, reduced proliferation, increased apoptosis	[78]
**2**	Ibuprofen and EGCG	In vitro (DU-145)	Prostate cancer	Better growth inhibition, reduced proliferation, increased apoptosis	[81]
**3**	Dasatinib and curcumin	In vitro (HCT-116) and in vivo (APCMin+/− mice)	Colon cancer	Increased inhibition of numerous metastatic processes, improved cell adhesion phenotype of HCT-116 colon cancer cells, regression of intestinal adenomas of 95%, reduced tumor progression, and improved apoptosis	[85]
**4**	Pterostilbene and EGCG	In vitro (MIA PaCa-2 and PANC-1)	Pancreatic cancer	Cell cycle arrest in S-phase in the case of MIA PaCa-2 cells, but not in PANC-1 cells, stimulated mitochondria depolarization and cytochrome-C upregulation in MIA PaCa-2 cells, but not in PANC-1 cells, enhanced antitumoral effects	[86]
**5**	Resveratrol and 5-fluorouracil	In vitro (TE-1 and A431) and in vivo (melanoma model)	Skin and esophageal cancer	Important tumor regression rate after four weeks of treatment, enhanced percentage of apoptotic cells and caspase-3, p53 and cleaved PARP levels, blocking cancer cells growth and induction of apoptosis	[97]

## Data Availability

Not applicable.
